# Modeling of noncovalent inhibitors of the papain-like protease (PLpro) from SARS-CoV-2 considering the protein flexibility by using molecular dynamics and cross-docking

**DOI:** 10.3389/fmolb.2024.1374364

**Published:** 2024-03-27

**Authors:** Jorge Luis Valdés-Albuernes, Erbio Díaz-Pico, Sergio Alfaro, Julio Caballero

**Affiliations:** Centro de Bioinformática, Simulación y Modelado (CBSM), Facultad de Ingeniería, Universidad de Talca, Talca, Chile

**Keywords:** papain-like protease, PLpro inhibitors, SARS-CoV-2, docking energy-activity correlation, flexible molecular docking, molecular dynamics

## Abstract

The papain-like protease (PLpro) found in coronaviruses that can be transmitted from animals to humans is a critical target in respiratory diseases linked to Severe Acute Respiratory Syndrome (SARS-CoV). Researchers have proposed designing PLpro inhibitors. In this study, a set of 89 compounds, including recently reported 2-phenylthiophenes with nanomolar inhibitory potency, were investigated as PLpro noncovalent inhibitors using advanced molecular modeling techniques. To develop the work with these inhibitors, multiple structures of the SARS-CoV-2 PLpro binding site were generated using a molecular sampling method. These structures were then clustered to select a group that represents the flexibility of the site. Subsequently, models of the protein-ligand complexes were created for the set of inhibitors within the chosen conformations. The quality of the complex models was assessed using LigRMSD software to verify similarities in the orientations of the congeneric series and interaction fingerprints to determine the recurrence of chemical interactions. With the multiple models constructed, a protocol was established to choose one per ligand, optimizing the correlation between the calculated docking energy values and the biological activities while incorporating the effect of the binding site’s flexibility. A strong correlation (R^2^ = 0.922) was found when employing this flexible docking protocol.

## 1 Introduction

At the end of 2019, the world was surprised with the appearance of pneumonia cases in Wuhan, China, caused by a new virus originating from bats (Hao et al., 2022). The causative virus, named SARS-CoV-2, spread rapidly worldwide, marking the onset of the first pandemic of the 21st century (Van Vo et al., 2021). It was determined that SARS-CoV-2 is a species of coronavirus from animals, similar to others that had previously caused similar diseases at the beginning of the century, serving as precursors and highlighting the profile posed by these types of viruses (Rota et al., 2003; Zhang et al., 2005; de Wit et al., 2016; Chafekar and Fielding, 2018). Consequently, researchers investigated viral infection mechanisms to identify treatment options for individuals affected by zoonotic coronaviruses (CoVs). These efforts yielded valuable insights, leading to the identification of molecular targets that are currently under investigation in the quest to develop specific drugs targeting CoVs.

When someone is infected with CoVs, certain proteins are produced that play important roles in the virus’s infection process. Two of these proteins, the proteases 3CLpro and PLpro, have been identified as responsible for processing large polyproteins encoded by the viral RNA genome (Lv et al., 2021; Yan and Wu, 2021; Hu et al., 2022). The functions of these two proteases have been identified; very conclusive information explains that PLpro plays a crucial role in coronavirus replication. It is involved in important biochemical processes such as deubiquitination and deISGylation of proteins in the host cells, which are vital for viral pathogenesis (Vere et al., 2022). Additionally, PLpro’s enzymatic activity antagonizes the host’s antiviral immune response, working in conjunction with viral protein processing (Shin et al., 2020).

PLpro has four distinct domains: the palm, the thumb, the fingers, and a terminal domain that resembles ubiquitin. The binding site of PLpro contains a catalytic triad composed of a cysteine, a histidine, and an aspartate (Cys111-His272-Asp286 in the SARS-CoV-2 PLpro), and integrates residues from the palm and thumb domains (Ratia et al., 2006). It also has specific subsites that can be occupied by the substrate RLRGG, which corresponds to the C-terminus of ubiquitin. The binding site can adopt both closed and open conformations, which are influenced by structural changes in the flexible blocking loop 2 (BL2). These changes modulate the recognition of different substrates (Henderson et al., 2020).

As PLpro is a cysteine protease with essential roles in the attack of coronaviruses (CoVs) on humans, it has become an important biological target (Brian Chia and Pheng Lim, 2023). Both covalent and non-covalent inhibitors have emerged for PLpro, many of which were reported between 2008 and 2014 (Ratia et al., 2008; Ghosh et al., 2009; Ghosh et al., 2010; Báez-Santos et al., 2014a), prior to the onset of the COVID-19 pandemic. These inhibitors were designed to target the PLpro identified in SARS-CoV-1. Among these inhibitors, the compound GRL-0617 emerged as a notable candidate due to its ability to inhibit viral replication *in vitro* of SARS-CoV-1 and SARS-CoV-2 viruses. Building upon this set of compounds and after the start of the COVID-19 pandemic, Shen and colleagues synthesized a new series of compounds derived from GRL-0617 with the intention of inhibiting SARS-CoV-2 PLpro (Shen et al., 2022).

The binding site of SARS-CoV-2 PLpro lacks sufficient space to accommodate ligands near the catalytic cysteine. To address this, GRL-0617 and its derivatives bind to the S_3_ and S_4_ subsites, causing an increase in space and a rearrangement of chemical groups, as confirmed by the opening of the BL2 loop. Crystallographic structures have been reported, enabling a comparison of these subsites in both the apo form and in the presence of some of these ligands. This helps identify which residues contribute to establishing a chemical match between specific ligands and the enzyme. This information serves as a valuable starting point for studying the family of 89 inhibitors synthesized and evaluated by Shen et al. (Shen et al., 2022) with the goal of finding a model that explains the greater activity of some compounds compared to others.

In a recent study, a computational analysis was conducted, successfully establishing a correlation between the activity of 67 non-covalent SARS-CoV-1 PLpro inhibitors and their energies, determined by docking while considering the flexibility of the binding sites (Castillo-Campos et al., 2023). Applying this methodology to the new inhibitory compounds of the latest PLpro variant will enable us to comprehend the intricacies involved in incorporating a larger number of compounds with greater structural variability. Our efforts have been focused on suggesting that the inclusion of significant flexibility in the binding site is crucial for developing models capable of predicting novel PLpro inhibitors.

## 2 Materials and Methods

### 2.1 Preparation of the studied ligands

The study by Shen et al. (Shen et al., 2022) provided information on a total of 89 chemical structures derived from GRL0617, along with their IC_50_ values. This reference was used to gather the data. Table 1 contains the chemical structures of each compound. The chemical structures were visualized using the Maestro Molecular Editor (version 12.8.117, Schrödinger LLC, New York, NY, United States, 2021) and processed using the LigPrep module within Maestro. The protonation states of the compounds were estimated using Epik (Shelley et al., 2007) at a physiological pH of 7.

**TABLE 1:** Structures and activities of SARS-CoV-2 PLpro inhibitors.

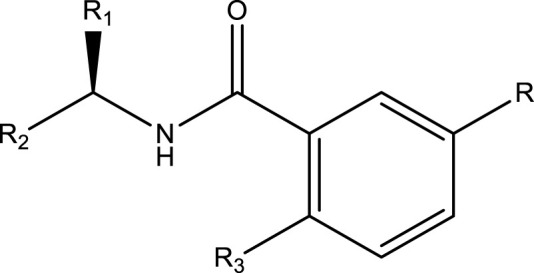							
Compound	R		R_1_	R_2_		R_3_	IC_50_ (µM)
GRL0617	‒NH_2_		Me	naphthalen-1-yl		Me	1.61
DY2-97	‒NH_2_		‒(CH_2_)_2_-OH	naphthalen-1-yl		Me	∼100
DY2-109	‒NH_2_		Me	naphthalen-1-yl		Br	21
DY2-137	(3-carboxyazetidin-1-yl)methyl		Me	naphthalen-1-yl		Me	3.3
DY2-138-2	(4-carboxypiperidin-1-yl)methyl		Me	naphthalen-1-yl		Me	6.1
DY2-139	Azetidin-3-yl(methyl)amino		Me	2-(thiophen-2-yl)phenyl		Me	>40
DY2-144	(Azetidin-3-ylamino)methyl		Me	naphthalen-1-yl		Me	1.3
DY2-149	Azetidin-3-yl(methyl)amino		Me	9*H*-carbazol-4-yl		Me	1.6
DY-2-153	Azetidin-3-ylamino		Me	9*H*-carbazol-4-yl		Me	1.8
DY-3-59	Azetidin-3-yl(methyl)amino		Me	benzo[*b*]thiophen-6-yl		Me	2.4
DY-3-66	Azetidin-3-yl(methyl)amino		Me	1-(cyclobutylmethyl)-1*H*-indol-4-yl		Me	3.3
XDY2-58	‒NH_2_		Me	1-methyl-1*H*-indol-3-yl		Me	<10
XDY2-62	‒NH_2_		Me	benzo[b]thiophen-3-yl		Me	3.3
XR8-8	Azetidin-3-ylamino		Me	3-(thiophen-3-yl)phenyl		Me	1.3
XR8-9	Azetidin-3-ylamino		Me	3-(1*H*-pyrrol-3-yl)phenyl		Me	1.8
XR8-14	Azetidin-3-ylamino		Me	3-(5-(piperazin-1-ylmethyl)thiophen-2-yl)phenyl		Me	1.2
XR8-15	Azetidin-3-ylamino		Me	3-(5-(morpholinomethyl)thiophen-2-yl)phenyl		Me	0.9
XR8-16	Azetidin-3-ylamino		Me	3-(5-(((1-methylpiperidin-4-yl)amino)methyl)thiophen-2-yl)phenyl		Me	1.6
XR8-17	Azetidin-3-ylamino		Me	3-(5-(((piperidin-4-ylmethyl)amino)methyl)thiophen-2-yl)phenyl		Me	2.7
XR8-23	Azetidin-3-ylamino		Me	3-(5-((cyclopentylamino)methyl)thiophen-2-yl)phenyl		Me	0.39
XR8-24	Azetidin-3-ylamino		Me	3-(5-(pyrrolidin-1-ylmethyl)thiophen-2-yl)phenyl		Me	0.56
XR8-30	Azetidin-3-ylamino		Me	3-(5-methylthiophen-2-yl)phenyl		Me	0.75
XR8-32-1	Azetidin-3-ylamino		Me	3-(5-carboxythiophen-2-yl)phenyl		Me	0.97
XR8-32-2	Azetidin-3-ylamino		Me	3-(5-(methoxycarbonyl)thiophen-2-yl)phenyl		Me	0.81
XR8-35	Azetidin-3-ylamino		Me	(*R*)-3-(5-(((tetrahydrofuran-2-yl)methyl)carbamoyl)thiophen-2-yl)phenyl		Me	0.92
XR8-38	Azetidin-3-ylamino		Me	(*S*)-3-(5-(((tetrahydrofuran-2-yl)methyl)carbamoyl)thiophen-2-yl)phenyl		Me	0.76
XR8-39	Azetidin-3-ylamino		Me	3-(5-(methylcarbamoyl)thiophen-2-yl)phenyl		Me	1.1
XR8-40	Azetidin-3-ylamino		Me	3-(5-((oxetan-2-ylmethyl)carbamoyl)thiophen-2-yl)phenyl		Me	0.82
XR8-49	Azetidin-3-ylamino		Me	(*R*)-3-(5-((3-hydroxypyrrolidin-1-yl)methyl)thiophen-2-yl)phenyl		Me	0.64
XR8-51	Azetidin-3-ylamino		Me	(*R*)-3-(5-((3-methylpyrrolidin-1-yl)methyl)thiophen-2-yl)phenyl		Me	1.1
XR8-56	Azetidin-3-ylamino		Me	3-(5-(azetidin-1-ylmethyl)thiophen-2-yl)phenyl		Me	2.2
XR8-57	Azetidin-3-ylamino		Me	(*S*)-3-(5-((3-hydroxypyrrolidin-1-yl)methyl)thiophen-2-yl)phenyl		Me	0.70
XR8-61	Azetidin-3-ylamino		Me	Br		Me	6.5
XR8-65	Azetidin-3-ylamino		Me	(*R*)- 3-(5-(((tetrahydrofuran-3-yl)amino)methyl)thiophen-2-yl)phenyl		Me	0.33
XR8-66	Azetidin-3-ylamino		Me	(*S*)- 3-(5-(((tetrahydrofuran-3-yl)amino)methyl)thiophen-2-yl)phenyl		Me	0.62
XR8-69	Azetidin-3-ylamino		Me	3-(5-(acetamidomethyl)thiophen-2-yl)phenyl		Me	0.37
XR8-77	Azetidin-3-ylamino		Me	3-(5-(((2-oxopyrrolidin-3-yl)amino)methyl)thiophen-2-yl)phenyl		Me	0.64
XR8-79	Azetidin-3-ylamino		Me	3-(5-(((5-oxopyrrolidin-3-yl)amino)methyl)thiophen-2-yl)phenyl		Me	0.41
XR8-83	Azetidin-3-ylamino		Me	3-(5-((((1*R*,3*S*)-3-hydroxycyclopentyl)amino)methyl)thiophen-2-yl)phenyl		Me	0.21
XR8-84	Azetidin-3-ylamino		Me	3-(5-((((1*R*,3*R*)-3-hydroxycyclopentyl)amino)methyl)thiophen-2-yl)phenyl		Me	0.43
XR8-89	Azetidin-3-ylamino		Me	3-(5-((((1*S*,3*R*)-3-hydroxycyclopentyl)amino)methyl)thiophen-2-yl)phenyl		Me	0.113
XR8-96	Azetidin-3-ylamino		Me	3-(5-((((1*S*,3*S*)-3-hydroxycyclopentyl)amino)methyl)thiophen-2-yl)phenyl		Me	0.25
XR8-98	‒NH_2_		Me	3-(5-(((*tert*-butoxycarbonyl)amino)methyl)thiophen-2-yl)phenyl		Me	0.81
XR8-101	‒NH_2_		Me	3-(5-(aminomethyl)thiophen-2-yl)phenyl		Me	1.8
XR8-103	‒NH_2_		Me	3-(5-(cyclopentanecarboxamidomethyl)thiophen-2-yl)phenyl		Me	1.1
XR8-104	‒NAc		Me	3-(5-(acetamidomethyl)thiophen-2-yl)phenyl		Me	2.3
XR8-106	‒NH_2_		Me	3-(5-((((1*S*,3*R*)-3-hydroxycyclopentyl)amino)methyl)thiophen-2-yl)phenyl		Me	1.4
YF4-134	‒NO_2_		Me	1*H*-indol-7-yl		Me	>10
YF4-136	‒NH_2_		Me	1*H*-indol-7-yl		Me	4.7
YF4-137	‒NH_2_		Me	1*H*-indol-3-yl		Me	∼100
ZN-2-180	(1-(*tert*-butoxycarbonyl)azetidin-3-yl)amino		Me	naphthalen-1-yl		Me	6.08
ZN-2-181	(1-(*tert*-butoxycarbonyl)piperidin-4-yl)amino		Me	naphthalen-1-yl		Me	1.1
ZN-2-182	1-(*tert*-butoxycarbonyl)azetidine-3-carboxamido		Me	naphthalen-1-yl		Me	5.5
ZN-2-183	1-(*tert*-butoxycarbonyl)piperidine-4-carboxamido		Me	naphthalen-1-yl		Me	6.0
ZN-2-184	Azetidin-3-ylamino		Me	naphthalen-1-yl		Me	1.01
ZN-2-185	Piperidin-4-ylamino		Me	naphthalen-1-yl		Me	0.6
ZN-2-186	Azetidine-3-carboxamido		Me	naphthalen-1-yl		Me	1.2
ZN-2-187	piperidine-4-carboxamido		Me	naphthalen-1-yl		Me	0.8
ZN-2-188-1	methyl(1-methylazetidin-3-yl)amino		Me	naphthalen-1-yl		Me	1.6
ZN-2-188-2	(1-methylazetidin-3-yl)amino		Me	naphthalen-1-yl		Me	4.3
ZN-2-189	(1-methylpiperidin-4-yl)amino		Me	naphthalen-1-yl		Me	0.7
ZN-2-190	‒NH_2_		Me	naphthalen-1-yl		F	>100
ZN-2-192	‒NH_2_		Me	naphthalen-1-yl		Cl	4.8
ZN-2-193	‒NH_2_		Me	naphthalen-1-yl		CF_3_	>10
ZN-2-197	Azetidin-3-yl(methyl)amino		Me	naphthalen-1-yl		Me	2.4
ZN-3-3	‒NH_2_		Me	naphthalen-1-yl		vinyl	>10
ZN-3-13	‒NH_2_		hydroxymethyl	naphthalen-1-yl		Me	∼100
ZN-3-19	‒NH_2_		Me	2-hydroxynaphthalen-1-yl		Me	>100
ZN-3-32	Azetidin-3-yl(methyl)amino		Me	2-hydroxynaphthalen-1-yl		Me	<100
ZN-3-33	Azetidin-3-yl(methyl)amino		Me	2-(azetidin-3-yloxy)naphthalen-1-yl		Me	>10
ZN-3-35	Azetidin-3-yl(methyl)amino		hydroxymethyl	naphthalen-1-yl		Me	>100
ZN-3-36	Azetidin-3-yl(methyl)amino		Me	isoquinolin-1-yl		Me	56
ZN-3-45	Azetidin-3-yl(methyl)amino		Me	1*H*-indol-7-yl		Me	5.7
ZN-3-56	((carboxymethyl)amino)methyl		Me	naphthalen-1-yl		Me	3.9
ZN-3-59	Azetidin-3-yl(methyl)amino		Me	benzo[*b*]thiophen-3-yl		Me	2.4
ZN-3-61	Azetidin-3-yl(methyl)amino		Et	naphthalen-1-yl		Me	>10
ZN-3-66	Azetidin-3-ylamino		Me	naphthalen-1-yl		Cl	4.1
ZN-3-67	Azetidin-3-ylamino		Me	benzo[*b*]thiophen-3-yl		Cl	8.5
ZN-3-70	Azetidin-3-yl(methyl)amino		Me	naphthalen-1-yl		Cl	10.7
ZN-3-71	Azetidin-3-yl(methyl)amino		Me	benzo[*b*]thiophen-3-yl		Cl	10.9
ZN-3-74	Azetidin-3-ylamino		Me	2-(thiophen-3-yl)phenyl		Cl	2.8
ZN-3-79	Azetidin-3-ylamino		Me	benzo[b]thiophen-3-yl		Me	1.9
ZN-3-80	Azetidin-3-ylamino		Me	2-(thiophen-3-yl)phenyl		Me	0.59
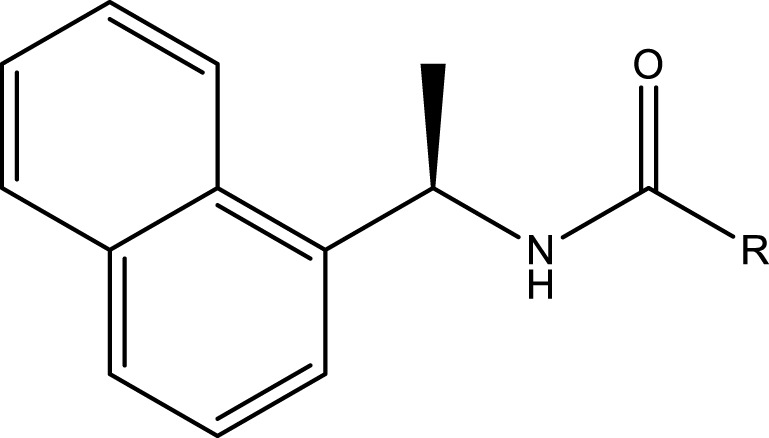							
Compound		R			IC_50_ (µM)		
DY2-115		2-amino-5-bromopyridin-4-yl			7		
DY-3-14		1*H*-indol-4-yl			>10		
DY-3-15		1*H*-indol-5-yl			0.8		
DY-3-65		5-methyl-1*H*-indol-4-yl			6.3		
DY-3-70		1-(azetidin-3-ylmethyl)-5-methyl-1*H*-indol-4-yl			6.4		
ZN-3-55		2-(azetidin-3-ylamino)-5-methylpyridin-4-yl			7.4		

### 2.2 Preparation of SARS-CoV-2 PLpro structures

We extracted the protein atomistic coordinates from crystal structures of the SARS-CoV-2 PLpro deposited in the Protein Data Bank (PDB). We specifically chose structures forming complexes with the inhibitors XR8-24 (7LBS), XR8-65 (7LOS), XR8-69 (7LLZ), XR8-83 (7LLF), and XR8-89 (7LBR) (Shen et al., 2022). When these PDB structures were compared, no appreciable variations were observed in the residues that constitute the S_3_ and S_4_ subsites. This determination was made through root-mean-square deviation (RMSD) calculations reported in the Supplementary Table S1.

PDB structures were prepared for docking calculations using the basic functionalities of Protein Preparation Wizard module (Schrödinger LLC, New York, NY, United States, 2021). This module removed crystallographic waters but retained the Zn^2+^, determined the neutrality or possible charge of protonatable groups, and performed a steepest descent restrained minimizations of the protein models using OPLS3 force field (Harder et al., 2016). During the minimization, heavy atoms were restrained with a harmonic potential of 25 kcal mol^−1^Å^−2^, hydrogens were left unrestrained, and heavy atoms were converged to RMSD = 0.3 Å.

### 2.3 Docking calculations

We applied docking to obtain ligand-PLpro models with Glide (Friesner et al., 2004). The studied compounds were docked within the spaces delimited between the residues at subsites S_3_-S_4_ of the PLpro structure with code 7LLF. A 20 × 20 × 20 Å^3^ grid with a grid spacing of 0.5 Å were defined. We utilized both the Glide standard (SP) and extra precision (XP) modes. Both modes together were employed to get high-quality solutions, although priority was given to the more rigorous solutions in XP mode (Friesner et al., 2006). The method was employed with parameters similar to those used in our previous study of naphthalene-derived inhibitors (Castillo-Campos et al., 2023). In the process of selecting the optimal solution for each ligand, we considered the ten best poses based on the most negative docking scoring energy values, coupled with a thorough comparison between these poses and those observed in the reference PDBs. The aforementioned comparison involved calculating RMSD values, as detailed in the following section, to evaluate consensus among binding modes of multiple docked ligands. Consequently, the best pose was determined by identifying the one with the most negative energy value and a reasonable orientation in line with the comparison between poses.

### 2.4 LigRMSD

When similar compounds are docked, we expect the way they bind to be similar to the binding modes of the compounds already co-crystallized in PLpro structures. Consequently, the ligands are also expected to have the same orientation when compared to each other. To confirm the similarity between orientations, we employ the LigRMSD method (Velázquez-Libera et al., 2020). LigRMSD calculates RMSD values by comparing two ligands that may be identical ‘or similar’ (non-identical). LigRMSD identifies common graphs (maximum common substructure) between the two ligands and compares the coordinates of the equal graphs, enabling the comparison of non-identical ligands. The match between graphs is defined using the values “%Ref” and “%Mol”. %Ref quantifies the percentage of overlapping structural elements between a docked compound and a selected reference, relative to the total number of atoms in that reference. On the other hand, %Mol quantifies the percentage of overlapping structural elements between a docked compound and a selected reference, relative to the total number of atoms in the docked compound. These values obtained from the LigRMSD server represent the maximum similarity between the compounds being compared, with high values of “%Ref” and “%Mol” associated with high similarity. If the two compared ligands are identical (%Ref = %Mol = 100), we perform the typical RMSD calculation to compare the coordinates between identical ligands. If the ligands are not identical, only the coordinates of the common graphs are compared, and in that case, %Ref and/or %Mol <100. Low values of %Ref and/or %Mol indicate that the comparison made is not appropriate, as it may involve comparing small portions between the two ligands.

Low RMSD values indicate that the orientations obtained for the ligands are similar, and high values of %Ref and %Mol indicate that the comparisons made were appropriate. LigRMSD operates in two modes: strict and flexible. In strict mode, the graphs must be composed of the same heavy atoms, while in flexible mode, atoms can be different in the graph (e.g., C can be changed for N). For example, when comparing benzene with pyridine, they are considered identical using the flexible mode.

Using LigRMSD, we compared the docking poses of the compounds XR8-24, XR8-65, XR8-69, XR8-83, and XR8-89 with their respective PDB structures 7LBS, 7LOS, 7LLZ, 7LLF, and 7LBR. Following that, we compared the docking poses of all the compounds with the docking poses obtained for GRL0617 and ZN-3-80. We selected these compounds as references because they possess specific characteristics compatible with different subsets of the entire dataset.

### 2.5 Interaction fingerprint (IFP)

Protein-ligand interaction fingerprints (IFPs) are simplified binary representations of the 3D structures of protein-ligand complexes (Deng et al., 2004). They encode whether specific interactions occur between the amino acids in the protein’s binding pocket and the ligand. The IFPs utilize a one-dimensional format to indicate the presence or absence of these interactions, providing a concise representation of the complex’s interaction patterns. IFPs were determined between the docked poses of ligands and residues in the SARS-CoV-2 PLpro binding site. Maestro’s Interaction Fingerprint panel was employed to generate them. The interaction matrix was constructed by including hydrophobic (H), polar (P), aromatic (Ar), hydrogen bond (HB) acceptor (A), HB donor (D), and charged (Ch) groups. An interaction was considered to occur when a PLpro residue was within a maximum cutoff distance of 4.0 Å from the ligand’s heavy atoms.

### 2.6 Gaussian accelerated molecular dynamics (GaMD) and correlation analysis

To ensure a diverse sampling of the SARS-CoV-2 PLpro binding site, molecular dynamics (MD) simulations were conducted. MD simulations are recognized because they can complement and enrich the information obtained from X-ray crystallography for proteins by considering solvation and obtaining velocity and positions of atoms over time (Amadei et al., 1993; Karplus and McCammon, 2002; Bakan et al., 2011; Rodríguez et al., 2011). In this way, they contribute to the understanding of the physics that allows the existence of the protein structure and to the understanding of its biological function. MD simulations were performed with ligands present at the binding site to keep the site open and allow for the inclusion of other ligands in subsequent cross-docking calculations. The structures with PDB IDs 7LBR and 7LLF were chosen as starting points (these PDB structures have better resolutions than their analogues). The compound XR8-89 with the highest binding affinity was kept in the binding site of both protein structures (obtained with docking as indicated in section 2.3). Both MD simulations were performed using the same ligand to ensure that the site was sampled under conditions as similar as possible. XR8-89, which is one of the largest ligands, was incorporated to ensure that each part of the site remained open for subsequent docking calculations.

Prior to the simulations, protein structures were processed with the Protein Preparation Wizard from Schrödinger LLC. The protein was immersed in a truncated octahedron of TIP3P waters, ensuring a separation of 12 Å or more from the box’s surface. The system was subjected to a steepest descent minimization procedure comprising 10,000 steps. For the equilibration, a first round involved heating the system to 310K over 1 ns using an isothermal-isovolumetric (NVT) assembly. After this, an 80-ns isothermal-isobaric (NPT) equilibration at 310K and 1 atm was done.

The pmemd. cuda implementation of Amber20 was used to apply Gaussian accelerated molecular dynamics (GaMD) (Miao and McCammon, 2017). In particular, we employed LiGaMD (Miao et al., 2020), designed for protein-ligand complexes. We conducted 60-ns MD simulations (with the same settings as the equilibration), with only the last 50 ns considered as the production phase. No atoms were restrained during all the MD simulations. The MD simulations are brief as its aim is to obtain a conformational variation in the active site, which is ensured by using the GaMD method. The resulting trajectories were analyzed using the VMD (Humphrey et al., 1996) and CPPTRAJ (Roe and Cheatham, 2013).

To capture a greater range of conformational diversity, the trajectories obtained for both systems were clustered using the K-means algorithm. An internal script utilizing the scikit-learn library (Varoquaux et al., 2015) was employed for this purpose. The clustering process considered six geometrical descriptors to define the clusters, including various distances between specific atoms within the PLpro binding site. These descriptors are: (a) RMSD value of Gln269, (b) distance between the more proximal carboxylate O of Asp164 and the amide N of Gln269, (c) distance between the hydroxyl O of Tyr268 and the amide N of Gln269, (d) distance between the amine N of Lys157 and the side chain O of Gln269, (e) distance between the backbone O of Asn267 and the N of Cys270, and (f) distance between the hydroxyl O of Tyr264 and the backbone O of Asn267. This process resulted in a dendrogram or “cluster tree,” where each leaf represented a single cluster and the root represented the largest cluster containing all sampled states.

Representative protein structures in the GaMD trajectories were identified with the clustering. Each of them was used as the protein in molecular docking calculations for each ligand, resulting in the generation of a set of poses for each ligand, each assigned with a docking energy value. These docking calculations were performed using the same parameters described in Section 2.3. Subsequently, we applied an in-house method programmed in Python (Muñoz-Gutierrez et al., 2016) to select, through a genetic algorithm (GA), the set of poses that best fits a maximum correlation between the docking energies and the experimental IC_50_ activities, converted to pIC_50_ values (pIC_50_ = -logIC_50_ with IC_50_ in M). In the GA search, random combinations were generated in each generation. The population underwent several genetic operations to produce the next generations, including one-point crossover and single-point mutation. The crossover probability was set to 0.6, and the mutation rate was set to 0.1. Elitism was also applied to prevent the loss of good combinations from one generation to the next, where the top 30% of combinations were retained.

Using this protocol, we obtained a set of models for the 89 inhibitor-PLpro complexes, allowing the analysis of conformational changes in the protein binding site necessary to incorporate ligands with interaction energies adjusted to their difference in experimental activity.

## 3 Results and discussion

### 3.1 Docking results

**FIGURE 1:**
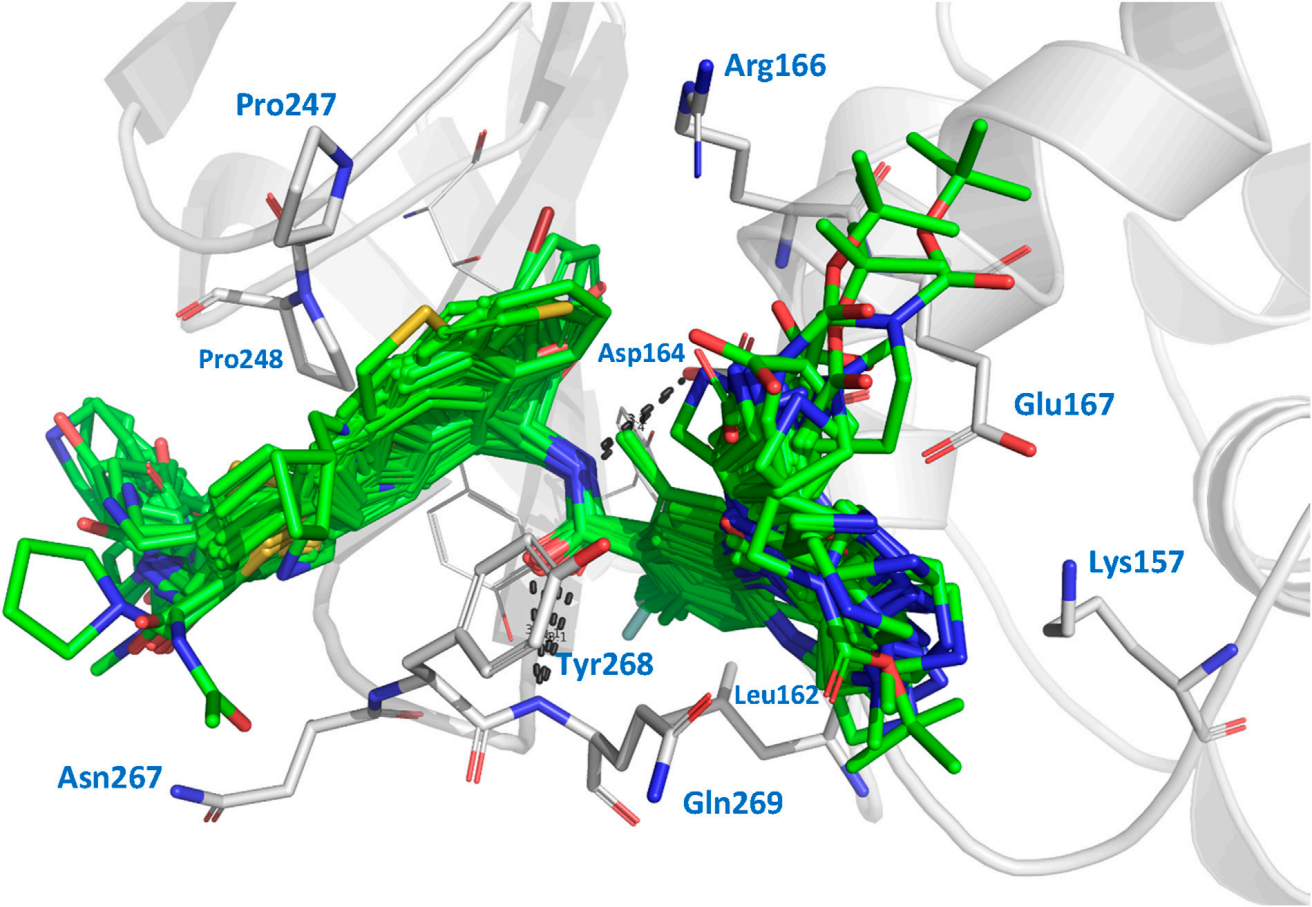
Docked structures within the SARS-CoV-2 PLpro binding site. Docked ligands are represented by green sticks. Relevant residues at protein subsites are represented by gray sticks. Hydrogens are omitted for clarity.

We performed docking calculations to investigate how the noncovalent inhibitors under study interact with the SARS-CoV-2 PLpro. The docking scoring energies can be found in the Supplementary Table S2. Our results revealed that all the ligands in the series share a common binding mode (Figure 1). Specifically, all the ligands preserve the hydrogen bonds (HBs) between the amide of the central scaffold and the residues Asp164 (side chain) and Gln269 (backbone NH), and position the R_2_ substituent at the S_4_ subsite and the R substituent (or large RR substituents) at the S_3_ subsite (the substituents are denoted as in Table 1). The naphthalen-1-yl substituents at R_2_ are oriented in two ways: most are oriented closer to Pro248 (referred to as the op2 orientation in the manuscript), while some are oriented closer to Pro247 (referred to as the op1 orientation). The compounds from series XR8, ZN-3-74, and ZN-3-80 oriented the 3-(thiophenyl)phenyl (and 3-(1*H*-pyrrolyl)phenyl in XR8-9) closer to Pro248 (op2 orientation). It was previously observed in crystallographic structures that the S_4_ subsite of PLpro is wide, and it was noted that substituents could adopt the two indicated orientations (Rut et al., 2020; Patchett et al., 2021). Regarding the S_3_ substituents, the vast majority of compounds oriented the R_3_ substituents near Leu162, while the R substituents were positioned close to the side chains of Asp167 and Asp164. The interactions described above align with the interactions previously reported in crystals containing compounds from series XR8 (Shen et al., 2022).

The comparison of the co-crystallized inhibitors with their respective docking poses yielded satisfactory results. For the comparison between the docking poses of compounds XR8-24, XR8-65, XR8-69, XR8-83, and XR8-89 with the poses in their respective crystals 7LBS, 7LOS, 7LLZ, 7LLF, and 7LBR (by aligning all the crystallographic structures with 7LLF), the RMSD values were 2.28, 2.47, 1.90, 2.56, and 1.75 Å, respectively. Comparisons between the docked structures and their references (as seen in Supplementary Figure S1) indicate that, although there are RMSD values greater than 2 Å, the orientations were adequate in all cases.

We also used the LigRMSD server (Velázquez-Libera et al., 2020) to assess the similarity in orientations among the 89 inhibitors. We used two references for the comparison, GRL0617 and ZN-3-80. Lower RMSD values indicate greater similarity between the compared poses. The results of these comparisons can be found in the Supplementary Table S3. Based on the %Ref and %Mol values, it was determined that the GRL0617 structure served as a more suitable reference for comparing the compounds of series DY2, XDY2, YF4, ZN-2, and ZN-3. On the other hand, ZN-3-80 is a more suitable reference for comparing the compounds of series XR8. The compounds ZN-3-45, ZN-3-59, ZN-3-67, ZN-3-71, ZN-3-74, and ZN-3-79 are exceptions, as they exhibit lower degree of similarity with both references, although they are closer in structure to ZN-3-80.

Compounds of series DY2, DY-3, ZN-2, and the majority of compounds from series ZN-3 exhibited values of %Ref (a measure of similarity to the reference) higher than 82.6% when GRL0617 is used as a reference. The RMSD values obtained were below 1.0 Å, with only five compounds (DY2-139, DY2-144, ZN-2-183, ZN-three to three, and ZN-3–33) showing RMSD values between 2.28 and 2.77 Å. In these five compounds, the R_2_ substituents (naphthalene in DY2-144, ZN-2-183, ZN-three to three, and ZN-3-33 and (*R*)-1-phenylethyl in DY2-139) oriented opposite to the naphthalene group in GRL0617, but their main scaffolds were correctly oriented. On the other hand, compounds of series XR8 exhibited values of %Ref higher than 82% when ZN-3-80 is used as a reference. The RMSD values obtained were between 0.30 and 2.39 Å. RMSD values around 2 Å were attributed to the diverse orientations of the R and R_2_ substituents within the subsites, providing ample space for various interaction possibilities. However, the main scaffold maintained a consistent orientation across all compounds (keeping the HBs with the residues Asp164 and Gln269). Notably, a distinct difference was observed in compounds XR8-83 and ZN-3-79, where the methyl group in R_3_ deviated from its position adjacent to Leu162. Therefore, from visual inspection and LigRMSD calculations, it is possible to conclude that the docking poses of all the ligands were aligned with the co-crystallized compounds and between them.

**FIGURE 2:**
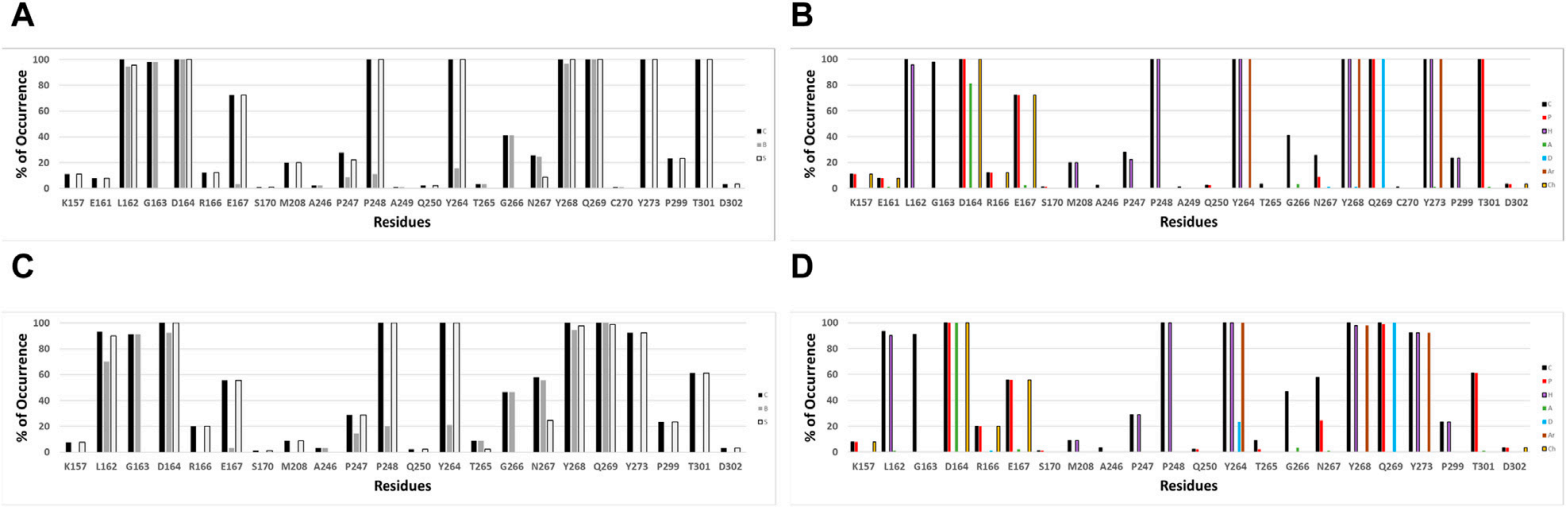
IFPs depicting the interactions between the docked compounds and SARS-CoV-2 PLpro. **(A, B)** Interactions of compounds with residues within the PLpro crystal identified as 7LLF. **(C, D)** Interactions observed in the selected complexes involving cross-docked compounds and SARS-CoV-2 PLpro conformations that exhibit the highest correlation. The graphs on the left side **(A, C)** illustrate the percentage of occurrence of contacts [C], interactions with the residue’s backbone [B], and interactions with the residue’s side chain [S]. The graphs on the right side **(B, D)** portray the occurrence percentages of diverse chemical interactions: contacts [C], polar [P], hydrophobic [H], HBs where the residue serves as an acceptor [A], HBs where the residue serves as a donor [D], aromatic [Ar], and electrostatic interactions involving charged groups [Ch].

IFPs were conducted to gain a deeper insight into the interactions between the docked ligands and PLpro. This analysis allows annotating the recurrent chemical interactions observed between the studied inhibitors and the protease binding site. In Figures 2A,B, it is possible to find the IFPs for the 89 compounds docked in the PLpro crystal with the code 7LLF. The polar interactions that connect the central amide group of the compounds with residues Asp164 and Gln269 are of great importance, as reflected in the IFPs. Asp164 is involved in polar interactions with all docked poses, occurring 100% of the time. It also acts as an HB acceptor, with a frequency of over 80%, reflecting its propensity to form HBs with the central amide NH group. The residue Gln269, located in the BL2 loop, exhibits polar contacts and serves as an HB donor in 100% of cases. Hydrophobic and aromatic interactions with residues Tyr264, Tyr268, and Tyr273 are consistently observed in all the docked structures. These residues collectively form an aromatic enclosure that contributes to the attraction and stabilization of the studied inhibitors. Specifically, Tyr268 plays a crucial role in closing the BL2 loop to adopt the closed conformation of the binding site (Báez-Santos et al., 2014b).

Other notable IFPs patterns are described as follows. Leu162 and Gly163 interacted with all ligands, while leucine’s side chain engaged in hydrophobic interactions with approximately 95% of the cases. Thr301 also contributed in all the cases with polar interactions. The residues Pro247 and Pro248 promote the occurrence of hydrophobic contacts at the interface between the protein and the ligand. Pro248 consistently exhibits hydrophobic contacts in 100% of cases, while Pro247 shows hydrophobic contributions with approximately 20% of occurrence. Glu167 participated in polar and charged contacts in approximately 72% of cases. Finally, Lys157 exhibited polar and charged interactions in around 10% of the docked structures.

### 3.2 Cross-docking results

To enhance the exploration of different chemical interactions and configurations within the binding site of the SARS-CoV-2 PLpro enzyme when forming complexes with the studied set of inhibitors, we conducted GaMD simulations following the procedures detailed in the Materials and Methods section. Specifically, two simulations were conducted on the solvated PDB structures with codes 7LBR and 7LLF in complex with XR8-89. We assessed the stability of these GaMD trajectories by measuring the RMSD of the positions of the backbone atoms of PLpro over time. The root mean square fluctuation (RMSF) values remained relatively consistent throughout the production simulations for all systems, as shown in Supplementary Figure S2.

Applying the clustering analysis to the GaMD simulations (as described in the Materials and Methods section), a set of twenty representative PLpro conformations was obtained, demonstrating structural diversity, and denoted as 7llf_0‒7llf_9 and 7lbr_0‒7lbr_9 in this manuscript. These conformations were useful for our subsequent cross-docking analysis, during which we docked the 89 compounds into these twenty PLpro structures, each exhibiting diverse binding site conformations. During the cross-docking process, we generated twenty different poses for each ligand. To validate the reliability of these poses, we employed LigRMSD (Velázquez-Libera et al., 2020) to ensure the presence of plausible solutions. Following this verification step, we identified representative complexes of PLpro and inhibitors for each ligand. This selection was carried out using a custom Python script developed *in-house* (Muñoz-Gutierrez et al., 2016). The script was designed to optimize the correlations between calculated docking energies and experimental PLpro inhibitory activities. It effectively identified a set of representative PLpro-inhibitor complexes that exhibited the strongest correlation between docking scores and experimental inhibitory activities. The energies associated with these complexes are provided in Supplementary Table S2.

**FIGURE 3:**
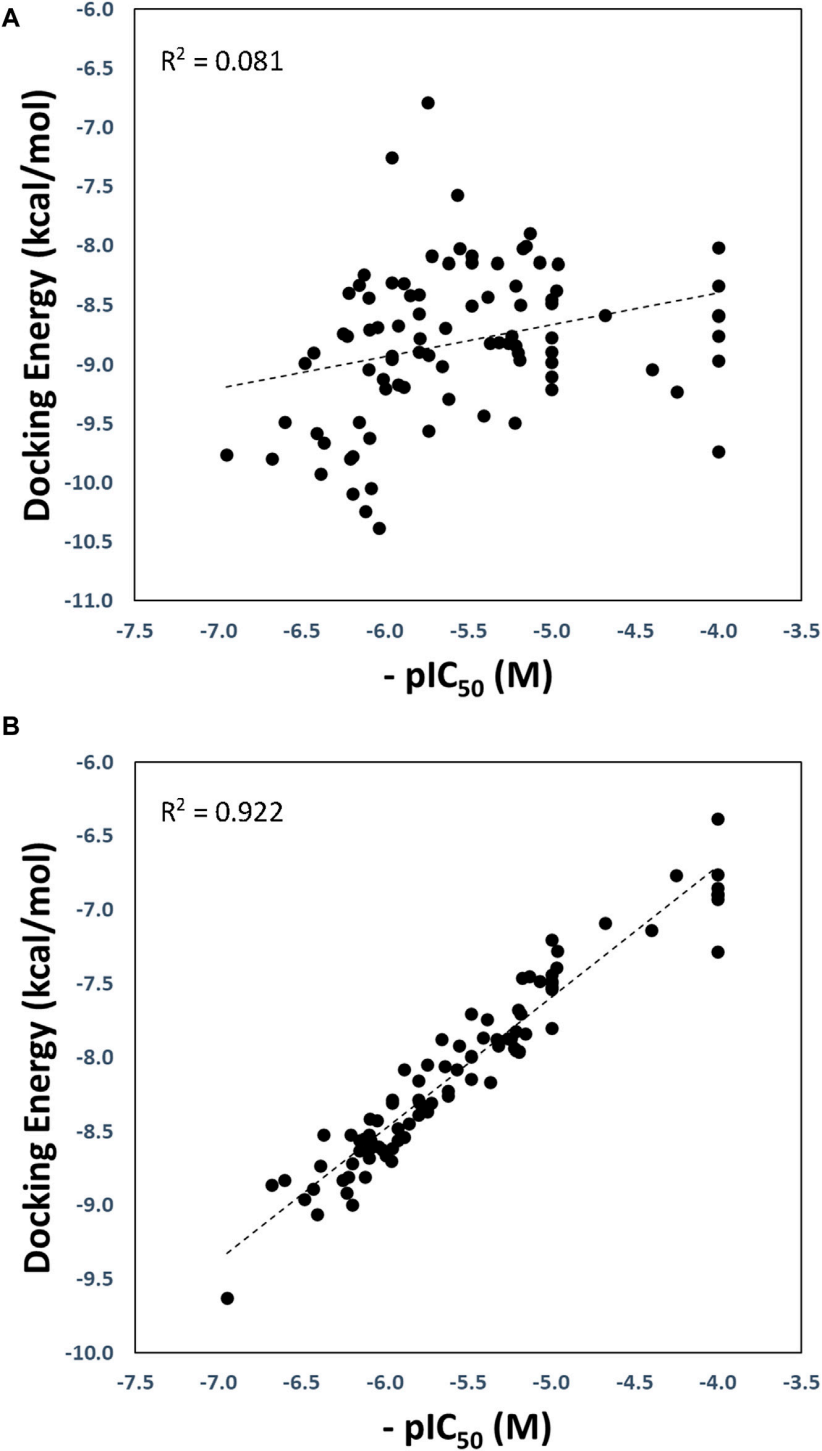
Regression plots of the docking scoring energies *versus* experimental activities (-pIC50) for the docking experiments performed in the SARS-CoV-2 PLpro structure with code 7LLF **(A)**, and for the cross-docking protocol **(B)**.

The optimized and reference correlations are presented in Figure 3. Notably, the correlation for the docking experiments conducted with structure with code 7LLF (used as reference) was found to be weak (R^2^ = 0.081; Figure 3A). This outcome aligns with expectations and is consistent with the known limitations of current docking scoring functions. It is well-known that scoring functions have demonstrated competence in docking and screening evaluations but may not excel in evaluating scoring power, which involves establishing a robust linear relationship between predicted and experimentally determined activities (Warren et al., 2006; Damm-Ganamet et al., 2013; Xu et al., 2015). To address this challenge, one strategy is to introduce flexibility into the protein binding site (Lexa and Carlson, 2012). In our approach, we harnessed various conformational states obtained through GaMD simulations, enabling flexibility within the binding site. As illustrated in Figure 3B, our method substantially improved the correlation between predicted and experimental pIC_50_ values, yielding an R^2^ value of 0.922.

The strong correlation observed signifies the effectiveness of our proposed protocol in elucidating the relationship between the molecular structure and activity. Within the dataset of twenty PLpro conformations, our script successfully identified fourteen protein conformations that combined different orientations of the residues in the binding site. These specific conformations are listed in Table 2, along with compounds docked into each PLpro conformation, crucial for generating the structure-activity relationship model with the highest R^2^ value.

**TABLE 2:** PLpro structures utilized in cross-docking experiments and the SARS-CoV-2 PLpro inhibitors involved in the structure-activity relationship model that exhibited the highest R^2^.

Protein conformation	Ligands	Protein conformation	Ligands
7llf_0	DY-2-153, DY-3-70, XR8-32-1, XR8-96, ZN-2-181, ZN-2-182, ZN-2-197, ZN-3-32, ZN-3-70, ZN-3-71, ZN-3-79	7lbr_0	DY2-97, XR8-16, XR8-23, XR8-51, XR8-104, YF4-134, ZN-3-55, ZN-3-74
7llf_1	XR8-17, XR8-57, YF4-136, ZN-2-185, ZN-2-190	7lbr_1	XR8-30, XR8-40, XR8-66, XR8-101, ZN-2-188-1, ZN-2-189, ZN-3-59
7llf_2	DY-3-65, XDY2-58, XR8-49, XR8-65, XR8-69, XR8-106, ZN-2-187, ZN-3-56	7lbr_2	DY-3-15, XR8-24, XR8-32-2, XR8-84, ZN-2-180
7llf_3	DY2-115, DY2-139, XR8-103, ZN-2-192, ZN-3-3, ZN-3-13, ZN-3-19, ZN-3-35, ZN-3-36, ZN-3-61	7lbr_5	DY2-137, DY-3-66, XDY2-62, XR8-38, XR8-39
7llf_5	DY2-109, DY2-138-2, XR8-8, XR8-56, YF4-137	7lbr_9	XR8-35, ZN-3-33
7llf_6	DY2-144, XR8-9, XR8-15, XR8-83, ZN-2-186, ZN-2-193		
7llf_7	DY2-149, DY-3-14, DY-3-59, XR8-14, XR8-77, XR8-79, XR8-89, ZN-2-188-2		
7llf_8	GRL0617, XR8-98, ZN-3-66, ZN-3-67, ZN-3-80		
7llf_9	XR8-61, ZN-2-183, ZN-2-184, ZN-3-45		

Our approach involved the application of GaMD simulations and clustering to generate diverse conformational states within the binding site of SARS-CoV-2 PLpro. The MD and clustering protocol generated variations in the binding site, enhancing its flexibility and producing new structural conformations that resulted in different PLpro configurations for subsequent cross-docking calculations. Since we obtained an excellent correlation between activities and docking energy values through the inclusion of these variations in the active site, it is worthwhile to examine what those variations were. Through this analysis, it was identified that the residues that had the most significant conformational changes were Arg166, Glu167, Met208 and Asn267.

**FIGURE 4:**
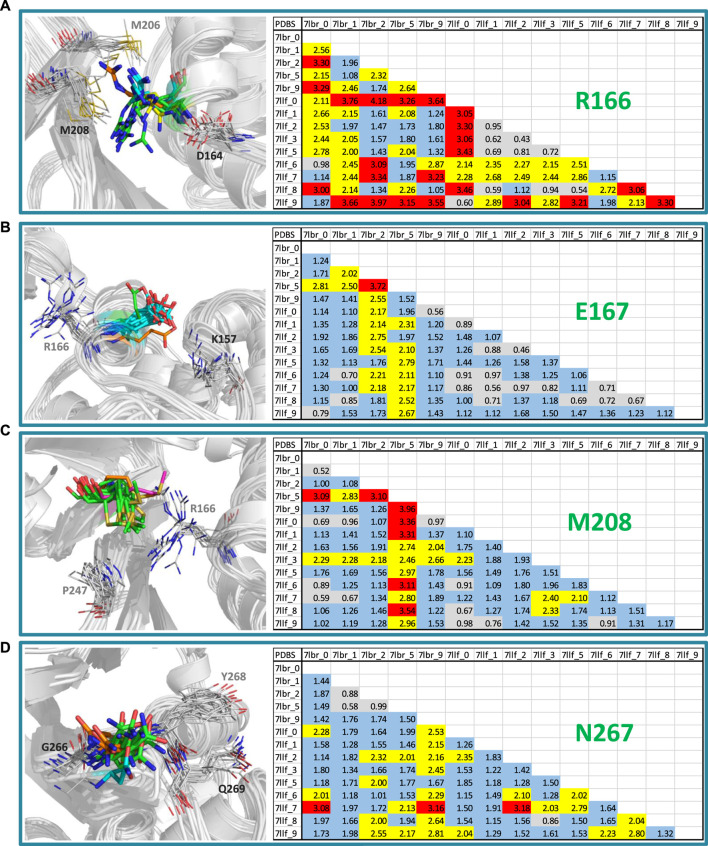
RMSD values (in Å) of the residues **(A)** Arg166, **(B)** Glu167, **(C)** Met208, and **(D)** Asn267, for the SARS-CoV-2 PLpro structures utilized in cross-docking experiments involved in the structure-activity relationship model that exhibited the highest R^2^. RMSD <1.0 Å are represented in gray, RMSD ≥1.0 Å and <2.0 Å are represented in blue, RMSD ≥2.0 Å and <3.0 Å are represented in yellow, and RMSD ≥3.0 Å are represented in red. Conformations of each residue are represented to the left, colored differently according to their orientations.

Figure 4 shows a visual inspection of these residues across the fourteen conformations present in the model that optimizes the structure-activity correlation. For Arg166, multiple orientations of the side chain guanidino group were observed (Figure 4A). In the majority of structures (7lbr_2, 7lbr_9, 7llf_1, 7llf_2, 7llf_3, 7llf_5, and 7llf_8), this group is directed towards the region where the ligand binds (depicted as green sticks). Conversely, in 7llf_0 and 7llf_9, it is positioned closer to Met206 (shown in orange sticks), while in 7lbr_1 and 7lbr_5, it is nearer to Asp164 (represented by yellow sticks). In 7llf_7, 7lbr_0, and 7llf_6, this group assumes a position equidistant between Met206 and Asp164. Glu167, next to Arg166, exhibited nearly consistent orientation across all structures (Figure 4B). However, there are two exceptions: in the 7lbr_2 structure, the side chain carboxylate group is directed toward Arg166 (depicted as green sticks), while in 7lbr_5, this group is oriented towards Lys157 (represented by orange sticks). Met208 also exhibits a relatively consistent orientation across structures between Arg166 and Pro247 (Figure 4C). However, there are two exceptions, in both structures 7llf_3 and 7lbr_5 the side chain methylthio group moves away from Pro247 (depicted as orange and purple sticks, respectively). Notably, structure 7lbr_5 (depicted in purple sticks) presents a notably different orientation compared to the rest. Finally, the most mobile residue within the BL2 loop appeared to be Asn267 (Figure 4D). The side chain of this residue did not exhibit significant differences in its orientations across structures. However, in 7llf_7, this residue showed the most notable difference from the remaining structures (illustrated by orange sticks), where its side chain moved away from the consecutive residue Tyr268. Additionally, in 7llf_9, Asn267 displayed a somewhat distinct orientation (depicted by cyan sticks), shifting its side chain away from both Tyr268 and the ligand binding site.

The conformational variations of the mentioned residues and those of others with less mobility, but with contributions to the flexibility of the binding site (the analyzes of K157 and Y268 are included in the Supplementary Figure S3) result in differences in the size and electrostatics of the active site. These differences significantly influence the variation in docking results and their energetic evaluation.

The validation of compound interactions within the SARS-CoV-2 PLpro binding site for protein-ligand complexes within the most highly correlated model was confirmed through IFPs. Earlier, the IFP analysis of docking-derived complexes highlighted key residues. These crucial residues would persist in the complexes obtained via cross-docking. IFPs for the selected complexes involving cross-docked compounds are reported in Figures 2C,D. When these IFPs are compared with those previously obtained considering a single structure (Figures 2A,B), it is possible to see that the interactions that characterize this series of complexes are maintained. It demonstrates the consistent chemical interactions between compounds and the PLpro binding site within the model showing the highest correlation. The significant interactions identified with residues Leu162, Gly163, Asp164, Glu167, Pro248, Tyr264, Tyr268, Gln269, Tyr273, and Thr301 were also evident in the IFPs within Figures 2C,D.

It is pertinent to compare the model obtained in this work for SARS-CoV-2 PLpro inhibitors with the model recently reported for naphthalene derivatives as SARS-CoV-1 PLpro inhibitors (Castillo-Campos et al., 2023). Both works were approached with the same methodology, with the only difference being that, in the current study, we decided to use shorter GaMD simulations to demonstrate the feasibility of applying the method with a brief sampling, which is particularly suitable when correlating a large number of compounds. Although the inhibitors studied in the previous work bind to the same binding site, their activities were evaluated against SARS-CoV-1 instead SARS-CoV-2 PLpro, making it impossible to unite all the compounds into a single model. The previous work was conducted for a smaller number of compounds, and their activities were generally lower. In that study, more than half of the compounds had IC_50_ activities >7 μM, while in the current work, only approximately one-third of the compounds have an activity higher than this value. Therefore, the data modeled in this work are more numerous and contain a greater number of compounds with favorable activities against PLpro. It should also be considered that the compounds in the current work exhibit more variability in the substituents placed on the S_4_ subsite, which may have influenced the greater number of PLpro conformations identified.

It is interesting to analyze whether we obtained conformations of SARS-CoV-2 PLpro prone to binding active ligands and others prone to binding inactive ligands. The former would be the best options for carrying out a virtual screening that detects new compounds, while the latter would be less recommended. Reviewing this detail in Table 2, it was observed that the 7llf_2, 7lbr_2, and 7llf_7 conformations bound six of the ten most active inhibitors (two each). On the other hand, the 7llf_3 conformation bound five of the ten least active inhibitors, while 7llf_5 bound two others. Based on these observations, it could be suggested that 7llf_3 (characterized by having M208 somewhat displaced, Figure 4C) would be the least recommended conformation for identifying new active compounds.

## 4 Conclusion

This study investigated a series of 89 noncovalent inhibitors of the PLpro enzyme from SARS-CoV-2. We utilized a flexible molecular docking protocol to analyze the structural features of the inhibitors and their interactions with the PLpro binding site. By incorporating protein flexibility and performing extensive docking simulations, we established a high correlation between the energy values obtained from the simulations and the experimental pIC_50_ values of the inhibitors. These findings contribute to developing potential drugs targeting PLpro in respiratory diseases caused by SARS-CoVs.

The research comprised of four steps:

i) Initially, the structures of the protein-ligand complexes were determined through a rigid docking approach; this involved predicting how the flexible ligands bind to a rigid protein,
ii) To explore the flexibility of the PLpro binding site, multiple protein conformations were generated using a short GaMD simulation; this allowed for a more comprehensive sampling of the different ways the binding site can adopt various shapes,
iii) A cross-docking experiment was conducted, which involved evaluating the interactions between the 89 compounds and selected PLpro conformations obtained in the previous step; this enabled to examine of how each compound interacts with the protein in various binding modes,
iv) From the cross-docking results, the protein-ligand complexes that have the highest correlation between the docking energies (the scoring energy values obtained from the docking simulations) and the experimental activities were selected; these selected complexes served as representative models, illustrating the most accurate relationship between the predicted docking energies and the experimental activities of the compounds.

As a result of this study, a collection of complexes was identified where the ligands interacted with a flexible binding site of PLpro. The methodology proposed in this research demonstrated its effectiveness, as evidenced by a correlation value of R^2^ = 0.922 obtained in the final step mentioned earlier. The inclusion of protein flexibility through the GaMD sampling of PDB structures played a pivotal role in achieving the intended objective. By considering the adaptability of the binding site, the limitations of a rigid docking approach, which fails to account for significant conformational changes in the protein upon ligand binding, were effectively overcome. Consequently, integrating flexibility in the binding site analysis presented a more rational and accurate approach.

In summary, the strategy employed in this article provides a robust framework for investigating PLpro ligands using computational tools. The approach reflects a potential conformational selection methodology, acknowledging the dynamic nature of ligand-protein interactions. Conducting a comprehensive structural investigation into the properties of SARS-CoV-2 PLpro inhibitors contributes positively to the field of research dedicated to the design and computational evaluation of more potent candidates targeting this protease.

## Data Availability

The original contributions presented in the study are included in the article/Supplementary Material, further inquiries can be directed to the corresponding author.

## References

[B1] Amadei A. Linssen A. B. Berendsen H. J. (1993). Essential dynamics of proteins. Proteins 17, 412–425. 10.1002/prot.340170408 8108382

[B2] Báez-Santos Y. M. Barraza S. J. Wilson M. W. Agius M. P. Mielech A. M. Davis N. M. (2014a). X-ray structural and biological evaluation of a series of potent and highly selective inhibitors of human coronavirus papain-like proteases. J. Med. Chem. 57, 2393–2412. 10.1021/jm401712t 24568342 PMC3983375

[B3] Báez-Santos Y. M. Mielech A. M. Deng X. Baker S. Mesecar A. D. (2014b). Catalytic function and substrate specificity of the papain-like protease domain of nsp3 from the Middle East respiratory syndrome coronavirus. J. Virol. 88, 12511–12527. 10.1128/JVI.01294-14 25142582 PMC4248884

[B4] Bakan A. Meireles L. M. Bahar I. (2011). ProDy: protein dynamics inferred from theory and experiments. Bioinformatics 27, 1575–1577. 10.1093/bioinformatics/btr168 21471012 PMC3102222

[B5] Brian Chia C. S. Pheng Lim S. (2023). A patent review on SARS coronavirus papain-like protease (PLpro) inhibitors. ChemMedChem 18, e202300216. 10.1002/cmdc.202300216 37248169

[B6] Castillo-Campos L. Velázquez-Libera J. L. Caballero J. (2023). Computational study of the binding orientation and affinity of noncovalent inhibitors of the papain-like protease (PLpro) from SARS-CoV-1 considering the protein flexibility by using molecular dynamics and cross-docking. Front. Mol. Biosci. 10, 1215499. 10.3389/fmolb.2023.1215499 37426421 PMC10326900

[B7] Chafekar A. Fielding B. C. (2018). MERS-CoV: understanding the latest human coronavirus threat. Viruses 10, 93. 10.3390/v10020093 29495250 PMC5850400

[B8] Damm-Ganamet K. L. Smith R. D. Dunbar J. B. Stuckey J. A. Carlson H. A. (2013). CSAR benchmark exercise 2011-2012: evaluation of results from docking and relative ranking of blinded congeneric series. J. Chem. Inf. Model 53, 1853–1870. 10.1021/ci400025f 23548044 PMC3753884

[B9] Deng Z. Chuaqui C. Singh J. (2004). Structural interaction fingerprint (SIFt): a novel method for analyzing three-dimensional protein-ligand binding interactions. J. Med. Chem. 47, 337–344. 10.1021/jm030331x 14711306

[B10] de Wit E. van Doremalen N. Falzarano D. Munster V. J. (2016). SARS and MERS: recent insights into emerging coronaviruses. Nat. Rev. Microbiol. 14, 523–534. 10.1038/nrmicro.2016.81 27344959 PMC7097822

[B11] Friesner R. A. Banks J. L. Murphy R. B. Halgren T. A. Klicic J. J. Mainz D. T. (2004). Glide: a new approach for rapid, accurate docking and scoring. 1. Method and assessment of docking accuracy. J. Med. Chem. 47, 1739–1749. 10.1021/jm0306430 15027865

[B12] Friesner R. A. Murphy R. B. Repasky M. P. Frye L. L. Greenwood J. R. Halgren T. A. (2006). Extra precision Glide: docking and scoring incorporating a model of hydrophobic enclosure for Protein−Ligand complexes. J. Med. Chem. 49, 6177–6196. 10.1021/jm051256o 17034125

[B13] Ghosh A. K. Takayama J. Aubin Y. Ratia K. Chaudhuri R. Baez Y. (2009). Structure-based design, synthesis, and biological evaluation of a series of novel and reversible inhibitors for the severe acute respiratory syndrome-coronavirus papain-like protease. J. Med. Chem. 52, 5228–5240. 10.1021/jm900611t 19645480 PMC3148848

[B14] Ghosh A. K. Takayama J. Rao K. V. Ratia K. Chaudhuri R. Mulhearn D. C. (2010). Severe acute respiratory syndrome coronavirus papain-like novel protease inhibitors: design, synthesis, protein-ligand X-ray structure and biological evaluation. J. Med. Chem. 53, 4968–4979. 10.1021/jm1004489 20527968 PMC2918394

[B15] Hao Y.-J. Wang Y.-L. Wang M.-Y. Zhou L. Shi J.-Y. Cao J.-M. (2022). The origins of COVID-19 pandemic: a brief overview. Transbound. Emerg. Dis. 69, 3181–3197. 10.1111/tbed.14732 36218169 PMC9874793

[B16] Harder E. Damm W. Maple J. Wu C. Reboul M. Xiang J. Y. (2016). OPLS3: a force field providing broad coverage of drug-like small molecules and proteins. J. Chem. Theory Comput. 12, 281–296. 10.1021/acs.jctc.5b00864 26584231

[B17] Henderson J. A. Verma N. Harris R. C. Liu R. Shen J. (2020). Assessment of proton-coupled conformational dynamics of SARS and MERS coronavirus papain-like proteases: implication for designing broad-spectrum antiviral inhibitors. J. Chem. Phys. 153, 115101. 10.1063/5.0020458 32962355 PMC7499820

[B18] Hu Q. Xiong Y. Zhu G.-H. Zhang Y.-N. Zhang Y.-W. Huang P. (2022). The SARS-CoV-2 main protease (Mpro): structure, function, and emerging therapies for COVID-19. MedComm 3, e151. 10.1002/mco2.151 35845352 PMC9283855

[B19] Humphrey W. Dalke A. Schulten K. (1996). VMD: visual molecular dynamics. J. Mol. Graph. 14, 33–38. 10.1016/0263-7855(96)00018-5 8744570

[B20] Karplus M. McCammon J. A. (2002). Molecular dynamics simulations of biomolecules. Nat. Struct. Biol. 9, 646–652. 10.1038/nsb0902-646 12198485

[B21] Lexa K. W. Carlson H. A. (2012). Protein flexibility in docking and surface mapping. Q. Rev. Biophys. 45, 301–343. 10.1017/S0033583512000066 22569329 PMC4272345

[B22] Lv Z. Cano K. E. Jia L. Drag M. Huang T. T. Olsen S. K. (2021). Targeting SARS-CoV-2 proteases for COVID-19 antiviral development. Front. Chem. 9, 819165. 10.3389/fchem.2021.819165 35186898 PMC8850931

[B23] Miao Y. Bhattarai A. Wang J. (2020). Ligand Gaussian accelerated molecular dynamics (LiGaMD): characterization of ligand binding thermodynamics and kinetics. J. Chem. Theory Comput. 16, 5526–5547. 10.1021/acs.jctc.0c00395 32692556 PMC7768792

[B24] Miao Y. McCammon J. A. (2017). Gaussian accelerated molecular dynamics: theory, implementation, and applications. Annu. Rep. Comput. Chem. 13, 231–278. 10.1016/bs.arcc.2017.06.005 29720925 PMC5927394

[B25] Muñoz-Gutierrez C. Adasme-Carreño F. Fuentes E. Palomo I. Caballero J. (2016). Computational study of the binding orientation and affinity of PPARγ agonists: inclusion of ligand-induced fit by cross-docking. RSC Adv. 6, 64756–64768. 10.1039/C6RA12084A

[B26] Patchett S. Lv Z. Rut W. Békés M. Drag M. Olsen S. K. (2021). A molecular sensor determines the ubiquitin substrate specificity of SARS-CoV-2 papain-like protease. Cell Rep. 36, 109754. 10.1016/j.celrep.2021.109754 34547223 PMC8423903

[B27] Ratia K. Pegan S. Takayama J. Sleeman K. Coughlin M. Baliji S. (2008). A noncovalent class of papain-like protease/deubiquitinase inhibitors blocks SARS virus replication. Proc. Natl. Acad. Sci. U.S.A. 105, 16119–16124. 10.1073/pnas.0805240105 18852458 PMC2571001

[B28] Ratia K. Saikatendu K. S. Santarsiero B. D. Barretto N. Baker S. C. Stevens R. C. (2006). Severe acute respiratory syndrome coronavirus papain-like protease: structure of a viral deubiquitinating enzyme. Proc. Natl. Acad. Sci. U.S.A. 103, 5717–5722. 10.1073/pnas.0510851103 16581910 PMC1458639

[B29] Rodríguez D. Piñeiro Á. Gutiérrez-de-Terán H. (2011). Molecular dynamics simulations reveal insights into key structural elements of adenosine receptors. Biochemistry 50, 4194–4208. 10.1021/bi200100t 21480628

[B30] Roe D. R. Cheatham T. E. (2013). PTRAJ and CPPTRAJ: software for processing and analysis of molecular dynamics trajectory data. J. Chem. Theory Comput. 9, 3084–3095. 10.1021/ct400341p 26583988

[B31] Rota P. A. Oberste M. S. Monroe S. S. Nix W. A. Campagnoli R. Icenogle J. P. (2003). Characterization of a novel coronavirus associated with severe acute respiratory syndrome. Science 300, 1394–1399. 10.1126/science.1085952 12730500

[B32] Rut W. Lv Z. Zmudzinski M. Patchett S. Nayak D. Snipas S. J. (2020). Activity profiling and crystal structures of inhibitor-bound SARS-CoV-2 papain-like protease: a framework for anti-COVID-19 drug design. Sci. Adv. 6, eabd4596. 10.1126/sciadv.abd4596 33067239 PMC7567588

[B33] Shelley J. C. Cholleti A. Frye L. L. Greenwood J. R. Timlin M. R. Uchimaya M. (2007). Epik: a software program for pK(a) prediction and protonation state generation for drug-like molecules. J. Comput. Aided Mol. Des. 21, 681–691. 10.1007/s10822-007-9133-z 17899391

[B34] Shen Z. Ratia K. Cooper L. Kong D. Lee H. Kwon Y. (2022). Design of SARS-CoV-2 PLpro inhibitors for COVID-19 antiviral therapy leveraging binding cooperativity. J. Med. Chem. 65, 2940–2955. 10.1021/acs.jmedchem.1c01307 34665619 PMC8547495

[B35] Shin D. Mukherjee R. Grewe D. Bojkova D. Baek K. Bhattacharya A. (2020). Papain-like protease regulates SARS-CoV-2 viral spread and innate immunity. Nature 587, 657–662. 10.1038/s41586-020-2601-5 32726803 PMC7116779

[B36] Van Vo G. Bagyinszky E. Park Y. S. Hulme J. An S. S. A. (2021). SARS-CoV-2 (COVID-19): beginning to understand a new virus. Adv. Exp. Med. Biol. 1321, 3–19. 10.1007/978-3-030-59261-5_1 33656709

[B37] Varoquaux G. Buitinck L. Louppe G. Grisel O. Pedregosa F. Mueller A. (2015). Scikit-learn: machine learning without learning the machinery. Getmob. Mob. Comp. Comm. 19, 29–33. 10.1145/2786984.2786995

[B38] Velázquez-Libera J. L. Durán-Verdugo F. Valdés-Jiménez A. Núñez-Vivanco G. Caballero J. (2020). LigRMSD: a web server for automatic structure matching and RMSD calculations among identical and similar compounds in protein-ligand docking. Bioinformatics 36, 2912–2914. 10.1093/bioinformatics/btaa018 31926012

[B39] Vere G. Alam M. R. Farrar S. Kealy R. Kessler B. M. O’Brien D. P. (2022). Targeting the ubiquitylation and ISGylation machinery for the treatment of COVID-19. Biomolecules 12, 300. 10.3390/biom12020300 35204803 PMC8869442

[B40] Warren G. L. Andrews C. W. Capelli A.-M. Clarke B. LaLonde J. Lambert M. H. (2006). A critical assessment of docking programs and scoring functions. J. Med. Chem. 49, 5912–5931. 10.1021/jm050362n 17004707

[B41] Xu W. Lucke A. J. Fairlie D. P. (2015). Comparing sixteen scoring functions for predicting biological activities of ligands for protein targets. J. Mol. Graph Model 57, 76–88. 10.1016/j.jmgm.2015.01.009 25682361

[B42] Yan S. Wu G. (2021). Spatial and temporal roles of SARS-CoV PLpro -A snapshot. FASEB J. 35, e21197. 10.1096/fj.202002271 33368679 PMC7883198

[B43] Zhang X. W. Yap Y. L. Danchin A. (2005). Testing the hypothesis of a recombinant origin of the SARS-associated coronavirus. Arch. Virol. 150, 1–20. 10.1007/s00705-004-0413-9 15480857 PMC7087341

