# Supplementary material for: Modeling of noncovalent inhibitors of the papain-like protease (PLpro) from SARS-CoV-2 considering the protein flexibility by using molecular dynamics and cross-docking
Source: Front Mol Biosci. 2024 Mar 27;11:1374364. doi: 10.3389/fmolb.2024.1374364 (PMC11004324; doi:10.3389/fmolb.2024.1374364)
Supplement: Supplementary file 1 [file DataSheet1.docx]

Supplementary Material

**Table S1.** RMSD values (Å) of SARS-CoV-2 PLpro residues within 5Å radius of the ligand atoms by using 7LBS as reference.^a^

| Residues | RMSD (Å) | | | |
| --- | --- | --- | --- | --- |
|  | 7LOS | 7LLZ | 7LLF | 7LBR |
| Tyr112 | 0.350 | 0.336 | 0.191 | 0.200 |
| Lys157 | 0.920 | 1.104 | 0.536 | 0.705 |
| Leu162 | 0.360 | 0.406 | 0.345 | 0.392 |
| Asp164 | 0.342 | 0.418 | 1.146 | 0.419 |
| Val165 | 0.261 | 0.250 | 0.247 | 0.206 |
| Arg166 | 0.558 | 0.623 | 0.274 | 0.610 |
| Glu167 | 0.851 | 0.528 | 0.334 | 0.677 |
| Met208 | 0.335 | 0.229 | 0.218 | 0.147 |
| Ala246 | 0.284 | 0.246 | 0.231 | 0.177 |
| Pro247 | 0.264 | 0.246 | 0.170 | 0.126 |
| Pro248 | 0.199 | 0.313 | 0.369 | 0.248 |
| Tyr264 | 1.400 | 1.396 | 1.391 | 1.383 |
| Gly266 | 0.173 | 0.388 | 0.418 | 0.352 |
| Asn267 | 0.797 | 0.965 | 1.268 | 0.909 |
| Tyr268 | 0.432 | 0.410 | 0.551 | 0.417 |
| Gln269 | 0.935 | 0.824 | 0.852 | 0.734 |
| Cys270 | 0.239 | 0.404 | 0.407 | 0.323 |
| Gly271 | 0.265 | 0.468 | 0.494 | 0.483 |
| Tyr273 | 0.258 | 0.198 | 0.305 | 0.187 |
| Thr301 | 0.412 | 0.366 | 0.347 | 0.313 |

**Table S2**. Activities of the studied compounds and their Glide scoring energies for docking performed in unique PDB structure and representative complexes from cross-docking.^a^

| Compounds | pIC_50_ | Glide scoring energy for docking in 7LLF (kcal/mol) | Glide scoring energy for representative complexes from cross-docking (kcal/mol) |
| --- | --- | --- | --- |
| GRL0617 | -5.79 | -8.78 | -8.31 |
| DY2-97 | -4.00 | -8.58 | -6.85 |
| DY2-109 | -4.68 | -8.58 | -7.09 |
| DY2-115 | -5.16 | -8.00 | -7.84 |
| DY2-137 | -5.48 | -8.50 | -7.70 |
| DY2-138-2 | -5.22 | -8.33 | -7.95 |
| DY2-139 | -4.40 | -9.04 | -7.14 |
| DY2-144 | -5.89 | -9.19 | -8.54 |
| DY2-149 | -5.80 | -8.90 | -8.15 |
| DY-2-153 | -5.74 | -9.56 | -8.04 |
| DY-3-14 | -5.00 | -8.89 | -7.50 |
| DY-3-15 | -6.10 | -9.04 | -8.68 |
| DY-3-59 | -5.17 | -8.02 | -7.46 |
| DY-3-65 | -5.20 | -8.90 | -7.68 |
| DY-3-66 | -5.48 | -8.13 | -7.99 |
| DY-3-70 | -5.19 | -8.96 | -7.96 |
| XDY2-58 | -5.00 | -8.45 | -7.80 |
| XDY2-62 | -5.48 | -8.08 | -8.14 |
| XR8-8 | -5.89 | -8.31 | -8.08 |
| XR8-9 | -5.74 | -8.92 | -8.36 |
| XR8-14 | -5.92 | -9.17 | -8.56 |
| XR8-15 | -6.05 | -8.68 | -8.42 |
| XR8-16 | -5.80 | -8.57 | -8.38 |
| XR8-17 | -5.57 | -7.57 | -8.08 |
| XR8-23 | -6.41 | -9.58 | -9.06 |
| XR8-24 | -6.25 | -8.74 | -8.83 |
| XR8-30 | -6.12 | -8.24 | -8.55 |
| XR8-32-1 | -6.01 | -9.12 | -8.62 |
| XR8-32-2 | -6.09 | -9.62 | -8.61 |
| XR8-35 | -6.04 | -10.38 | -8.60 |
| XR8-38 | -6.12 | -10.24 | -8.81 |
| XR8-39 | -5.96 | -8.31 | -8.28 |
| XR8-40 | -6.09 | -10.05 | -8.56 |
| XR8-49 | -6.19 | -9.78 | -8.72 |
| XR8-51 | -5.96 | -7.25 | -8.61 |
| XR8-56 | -5.66 | -9.02 | -7.88 |
| XR8-57 | -6.15 | -9.48 | -8.63 |
| XR8-61 | -5.19 | -8.50 | -7.71 |
| XR8-65 | -6.48 | -8.99 | -8.96 |
| XR8-66 | -6.21 | -9.80 | -8.52 |
| XR8-69 | -6.43 | -8.90 | -8.89 |
| XR8-77 | -6.19 | -10.10 | -9.00 |
| XR8-79 | -6.39 | -9.93 | -8.73 |
| XR8-83 | -6.68 | -9.80 | -8.86 |
| XR8-84 | -6.37 | -9.66 | -8.52 |
| XR8-89 | -6.95 | -9.76 | -9.63 |
| XR8-96 | -6.60 | -9.49 | -8.83 |
| XR8-98 | -6.09 | -8.70 | -8.41 |
| XR8-101 | -5.74 | -6.79 | -8.34 |
| XR8-103 | -5.96 | -8.95 | -8.30 |
| XR8-104 | -5.64 | -8.69 | -8.06 |
| XR8-106 | -5.85 | -8.41 | -8.45 |
| YF4-134 | -5.00 | -8.48 | -7.52 |
| YF4-136 | -5.33 | -8.14 | -7.88 |
| YF4-137 | -4.00 | -8.02 | -6.76 |
| ZN-2-180 | -5.22 | -8.84 | -7.82 |
| ZN-2-181 | -5.96 | -8.92 | -8.70 |
| ZN-2-182 | -5.26 | -8.82 | -7.87 |
| ZN-2-183 | -5.22 | -9.49 | -7.94 |
| ZN-2-184 | -6.00 | -9.20 | -8.66 |
| ZN-2-185 | -6.22 | -8.39 | -8.81 |
| ZN-2-186 | -5.92 | -8.67 | -8.48 |
| ZN-2-187 | -6.10 | -8.43 | -8.52 |
| ZN-2-188-1 | -5.80 | -8.40 | -8.28 |
| ZN-2-188-2 | -5.37 | -8.82 | -8.17 |
| ZN-2-189 | -6.16 | -8.32 | -8.56 |
| ZN-2-190 | -4.00 | -8.76 | -7.28 |
| ZN-2-192 | -5.32 | -8.81 | -7.92 |
| ZN-2-193 | -5.00 | -8.98 | -7.20 |
| ZN-2-197 | -5.62 | -9.29 | -8.22 |
| ZN-3-3 | -5.00 | -8.77 | -7.44 |
| ZN-3-13 | -4.00 | -8.97 | -6.90 |
| ZN-3-19 | -4.00 | -8.33 | -6.93 |
| ZN-3-32 | -4.00 | -8.59 | -6.90 |
| ZN-3-33 | -5.00 | -9.21 | -7.54 |
| ZN-3-35 | -4.00 | -9.74 | -6.38 |
| ZN-3-36 | -4.25 | -9.23 | -6.77 |
| ZN-3-45 | -5.24 | -8.76 | -7.87 |
| ZN-3-55 | -5.13 | -7.90 | -7.45 |
| ZN-3-56 | -5.41 | -9.43 | -7.87 |
| ZN-3-59 | -5.62 | -8.14 | -8.26 |
| ZN-3-61 | -5.00 | -9.10 | -7.48 |
| ZN-3-66 | -5.39 | -8.43 | -7.74 |
| ZN-3-67 | -5.07 | -8.14 | -7.48 |
| ZN-3-70 | -4.97 | -8.37 | -7.39 |
| ZN-3-71 | -4.96 | -8.15 | -7.28 |
| ZN-3-74 | -5.55 | -8.02 | -7.92 |
| ZN-3-79 | -5.72 | -8.08 | -8.30 |
| ZN-3-80 | -6.23 | -8.76 | -8.92 |

^a^ Glide scoring energies for cross-docking reflect the interactions between compounds and the conformations used as receptors for cross-docking (pairs of PLpro conformations extracted from GaMD and each compound are defined in Table 2).

**Figure S1.** Alignment of SARS-CoV-2 PLpro inhibitor docked structures on inhibitor X-ray reference structures. (A) Compound XR8-24 (PDB: 7LBS); (B) compound XR8-65 (PDB: 7LOS); (C) compound XR8-69 (PDB: 7LLZ), (D) compound XR8-83 (PDB: 7LLF); (E) compound XR8-89 (PDB: 7LBR). Crystal ligand structures are represented in orange, and docking results are represented in green. RMSD values are reported.


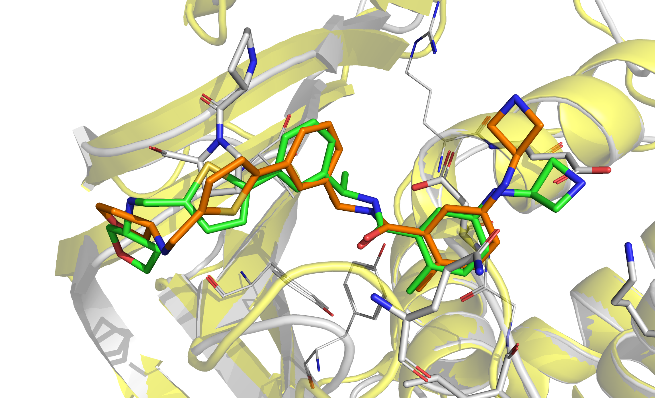


**RMSD = 2.47 Å**

**B**


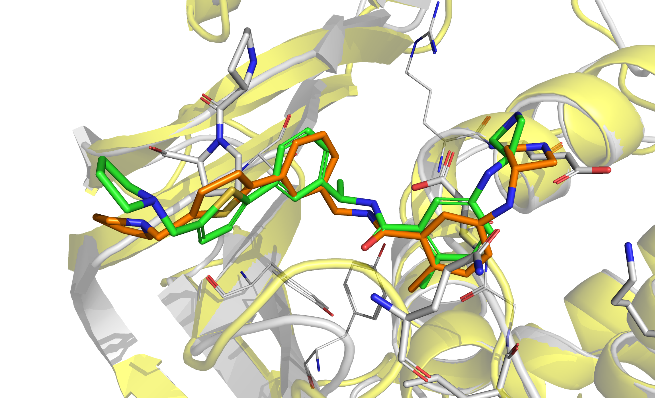


**RMSD = 2.28 Å**

**A**


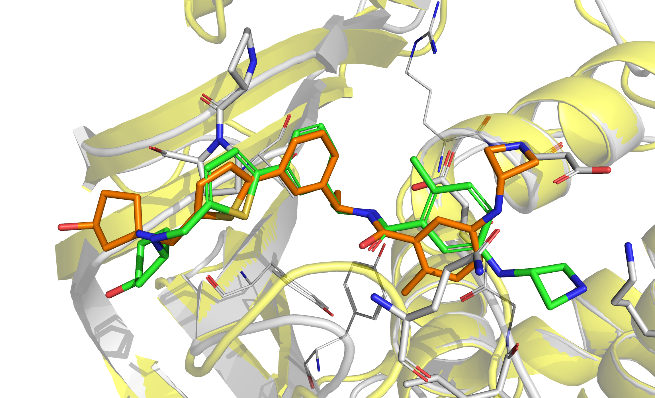


**RMSD = 2.56 Å**

**D**


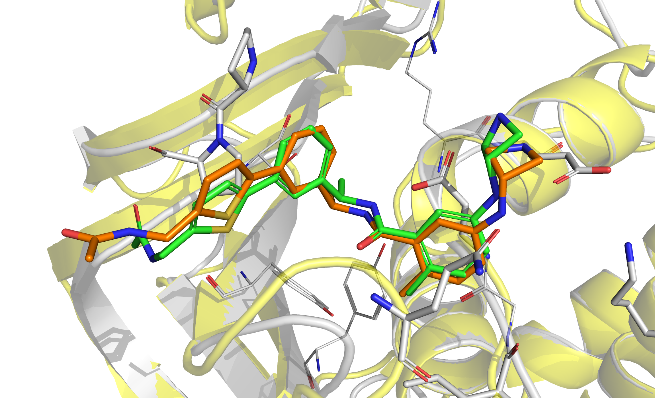


**RMSD = 1.90 Å**

**C**


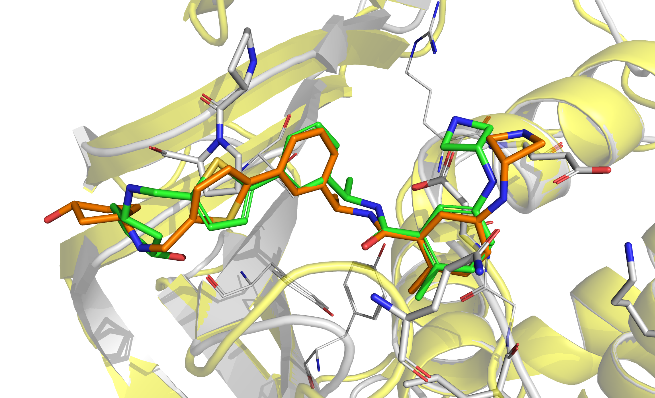


**RMSD = 1.75 Å**

**E**

**Table S3.** RMSD values of the obtained docking pose common fragments for the studied compounds with respect to the docking results of GRL0617 and ZN-3-80 inside the SARS-CoV-2 PLpro.

|  | Reference compound GRL0617 | | | Reference compound ZN-3-80 | | |
| --- | --- | --- | --- | --- | --- | --- |
| Compounds | RMSD (Å) ^a^ | %Ref ^b^ | %Mol ^c^ | RMSD (Å) ^a^ | %Ref ^b^ | %Mol ^c^ |
| DY2-97 | **0.84** | **100.00** | **92.00** | 0.82 | 67.86 | 76.00 |
| DY2-109 | **0.26** | **95.65** | **95.65** | 1.16 | 64.29 | 78.26 |
| DY2-115* | **0.77** ^d^ | **100.00** ^d^ | **100.00** ^d^ | 0.77 ^d^ | 67.86 ^d^ | 82.61 ^d^ |
| DY2-137 | **0.37** | **95.65** | **73.33** | 2.28 ^d^ | 82.14 ^d^ | 76.67 ^d^ |
| DY2-138-2 | **0.30** | **95.65** | **68.75** | 1.17 ^d^ | 71.43 ^d^ | 62.50 ^d^ |
| DY2-139 | **2.28** | **82.61** | **65.52** | 3.22 | 82.14 | 79.31 |
| DY2-144 | **2.57** | **95.65** | **78.57** | 0.58 | 64.29 | 64.29 |
| DY2-149 | **0.62** | **82.61** | **61.29** | 1.40 | 82.14 | 74.19 |
| DY-2-153 | **0.59** | **82.61** | **63.33** | 1.31 | 82.14 | 76.67 |
| DY-3-14 | **0.56** | **91.30** | **87.50** | 0.72 | 60.71 | 70.83 |
| DY-3-15 | **0.38** | **91.30** | **87.50** | 0.96 | 60.71 | 70.83 |
| DY-3-59 | 0.35 | 82.61 | 70.37 | **1.14** | **82.14** | **85.19** |
| DY-3-65 | **0.94** | **95.65** | **88.00** | 1.55 | 64.29 | 72.00 |
| DY-3-66 | 0.27 | 82.61 | 59.38 | **1.08** | **82.14** | **71.88** |
| DY-3-70 | **0.81** | **95.65** | **73.33** | 1.50 | 64.29 | 60.00 |
| XDY2-58 | **0.12** | **60.87** | **60.87** | 0.61 | 50.00 | 60.87 |
| XDY2-62 | **0.46** | **60.87** | **63.64** | 0.52 | 50.00 | 63.64 |
| XR8-8 | 1.03 | 82.61 | 67.86 | **0.87** ^d^ | **100.00** ^d^ | **100.00** ^d^ |
| XR8-9 | 1.13 | 82.61 | 67.86 | **1.93** ^d^ | **100.00** ^d^ | **100.00** ^d^ |
| XR8-14 | 1.23 | 82.61 | 54.29 | **1.74** | **100.00** | **80.00** |
| XR8-15 | 1.15 | 82.61 | 54.29 | **2.33** | **100.00** | **80.00** |
| XR8-16 | 0.73 | 82.61 | 51.35 | **1.06** | **100.00** | **75.68** |
| XR8-17 | 1.11 | 82.61 | 51.35 | **2.34** | **100.00** | **75.68** |
| XR8-23 | 0.63 | 82.61 | 54.29 | **0.79** | **100.00** | **80.00** |
| XR8-24 | 1.25 | 82.61 | 55.88 | **2.24** | **100.00** | **82.35** |
| XR8-30 | 1.20 | 82.61 | 65.52 | **2.27** | **100.00** | **96.55** |
| XR8-32-1 | 1.12 | 82.61 | 61.29 | **1.13** | **100.00** | **90.32** |
| XR8-32-2 | 0.76 | 82.61 | 59.38 | **1.03** | **100.00** | **87.50** |
| XR8-35 | 0.74 | 82.61 | 51.35 | **1.92** | **100.00** | **75.68** |
| XR8-38 | 0.79 | 82.61 | 51.35 | **1.03** | **100.00** | **75.68** |
| XR8-39 | 0.89 | 82.61 | 59.38 | **1.00** | **100.00** | **87.50** |
| XR8-40 | 0.98 | 82.61 | 52.78 | **2.01** | **100.00** | **77.78** |
| XR8-49 | 0.99 | 82.61 | 54.29 | **1.09** | **100.00** | **80.00** |
| XR8-51 | 1.24 | 82.61 | 54.29 | **2.11** | **100.00** | **80.00** |
| XR8-56 | 1.24 | 82.61 | 57.58 | **1.70** | **100.00** | **84.85** |
| XR8-57 | 0.96 | 82.61 | 54.29 | **0.85** | **100.00** | **80.00** |
| XR8-61 | 0.66 | 82.61 | 79.17 | **2.35** | **82.14** | **95.83** |
| XR8-65 | 1.08 | 82.61 | 54.29 | **1.03** | **100.00** | **80.00** |
| XR8-66 | 0.61 | 82.61 | 54.29 | **1.09** | **100.00** | **80.00** |
| XR8-67 | 0.72 | 82.61 | 52.78 | **2.16** | **100.00** | **77.78** |
| XR8-69 | 1.05 | 82.61 | 57.58 | **1.94** | **100.00** | **84.85** |
| XR8-77 | 1.22 | 82.61 | 52.78 | **2.09** | **100.00** | **77.78** |
| XR8-79 | 0.97 | 82.61 | 52.78 | **0.97** | **100.00** | **77.78** |
| XR8-83 | 2.09 | 82.61 | 52.78 | **1.47** | **100.00** | **77.78** |
| XR8-84 | 0.72 | 82.61 | 52.78 | **2.16** | **100.00** | **77.78** |
| XR8-89 | 0.99 | 82.61 | 52.78 | **1.93** | **100.00** | **77.78** |
| XR8-96 | 1.01 | 82.61 | 52.78 | **2.39** | **100.00** | **77.78** |
| XR8-98 | 1.20 | 82.61 | 57.58 | **1.15** | **85.71** | **72.73** |
| XR8-101 | 1.10 | 82.61 | 73.08 | **1.03** | **85.71** | **92.31** |
| XR8-103 | 1.09 | 82.61 | 57.58 | **1.00** | **85.71** | **72.73** |
| XR8-104 | 0.88 | 82.61 | 59.38 | **0.30** | **85.71** | **75.00** |
| XR8-106 | 0.56 | 82.61 | 59.38 | **0.58** | **85.71** | **75.00** |
| YF4-134 | **0.16** | **82.61** | **79.17** | 0.97 | 67.86 | 79.17 |
| YF4-136 | **0.74** | **82.61** | **86.36** | 1.61 | 67.86 | 86.36 |
| YF4-137 | **0.70** | **60.87** | **63.64** | 0.60 | 50.00 | 63.64 |
| ZN-2-180 | **0.73** | **100.00** | **67.65** | 1.92 | 82.14 | 67.65 |
| ZN-2-181 | **0.77** | **100.00** | **63.89** | 1.05 | 71.43 | 55.56 |
| ZN-2-182 | **0.25** | **100.00** | **63.89** | 0.96 | 67.86 | 52.78 |
| ZN-2-183 | **2.39** | **100.00** | **60.53** | 0.73 | 67.86 | 50.00 |
| ZN-2-184 | **0.18** | **100.00** | **85.19** | 1.94 | 82.14 | 85.19 |
| ZN-2-185 | **0.75** | **100.00** | **79.31** | 1.43 | 71.43 | 68.97 |
| ZN-2-186 | **0.17** | **100.00** | **79.31** | 0.93 | 67.86 | 65.52 |
| ZN-2-187 | **0.35** | **100.00** | **74.19** | 1.12 | 67.86 | 61.29 |
| ZN-2-188-1 | **0.28** | **100.00** | **79.31** | 1.09 | 82.14 | 79.31 |
| ZN-2-188-2 | **0.20** | **100.00** | **82.14** | 1.11 | 82.14 | 82.14 |
| ZN-2-189 | **0.75** | **100.00** | **76.67** | 0.98 | 71.43 | 66.67 |
| ZN-2-190 | **0.39** | **95.65** | **95.65** | 1.26 | 64.29 | 78.26 |
| ZN-2-192 | **0.14** | **95.65** | **95.65** | 1.04 | 64.29 | 78.26 |
| ZN-2-193 | **0.55** | **100.00** | **88.46** | 0.96 | 67.86 | 73.08 |
| ZN-2-197 | **0.54** | **100.00** | **82.14** | 2.01 | 82.14 | 82.14 |
| ZN-3-3 | **2.52** | **100.00** | **95.83** | 0.84 | 67.86 | 79.17 |
| ZN-3-13 | **0.99** | **100.00** | **95.83** | 1.53 | 67.86 | 79.17 |
| ZN-3-19 | **0.75** | **100.00** | **95.83** | 0.95 | 67.86 | 79.17 |
| ZN-3-32 | **0.33** | **100.00** | **79.31** | 0.88 | 82.14 | 79.31 |
| ZN-3-33 | **2.77** | **100.00** | **69.70** | 2.21 | 82.14 | 69.70 |
| ZN-3-35 | **0.64** | **100.00** | **79.31** | 1.21 | 82.14 | 79.31 |
| ZN-3-36* | **0.99** ^d^ | **100.00** ^d^ | **82.14** ^d^ | 1.53 ^d^ | 82.14 ^d^ | 82.14 ^d^ |
| ZN-3-45 | 0.64 | 82.61 | 70.37 | **2.44** | **82.14** | **85.19** |
| ZN-3-55* | **0.70** ^d^ | **100.00** ^d^ | **85.19** ^d^ | 2.12 ^d^ | 82.14 ^d^ | 85.19 ^d^ |
| ZN-3-56 | **0.18** | **95.65** | **78.57** | 1.00 | 64.29 | 64.29 |
| ZN-3-59 | 0.31 | 60.87 | 51.85 | **0.79** | **64.29** | **66.67** |
| ZN-3-61 | **0.74** | **100.00** | **79.31** | 2.34 | 82.14 | 79.31 |
| ZN-3-66 | **0.60** | **95.65** | **81.48** | 2.22 | 78.57 | 81.48 |
| ZN-3-67 | 0.58 | 56.52 | 50.00 | **2.49** | **60.71** | **65.38** |
| ZN-3-70 | **0.29** | **95.65** | **78.57** | 1.17 | 78.57 | 78.57 |
| ZN-3-71 | 0.34 | 56.52 | 48.15 | **2.50** | **60.71** | **62.96** |
| ZN-3-74 | 1.12 | 78.26 | 64.29 | **1.11** | **96.43** | **96.43** |
| ZN-3-79 | 2.39 | 60.87 | 53.85 | **2.24** | **64.29** | **69.23** |
| GRL0617 |  |  |  | 0.98 | 67.86 | 82.61 |
| ZN-3-80 | 0.98 | 82.61 | 67.86 |  |  |  |

a RMSD values considering only the common chemical fragments between the docked compound and the defined reference compound.

b %RefMatch refers to the percent of common graphs between the docked and reference compound concerning the total number of atoms of the reference compound.

c %MolMatch refers to the percent of common graphs between the docked and reference compound regarding the total number of atoms of the docked compound.

d In this case, difference in ring heavy atoms were not considered between the docked compound and the reference compound (flexible mode in LigRMSD).

**Figure S2.** Root mean square fluctuation (RMSF) values of the residues in the binding site comparing the conformations of SARS-CoV-2 PLpro that participated in the model with optimal correlation.


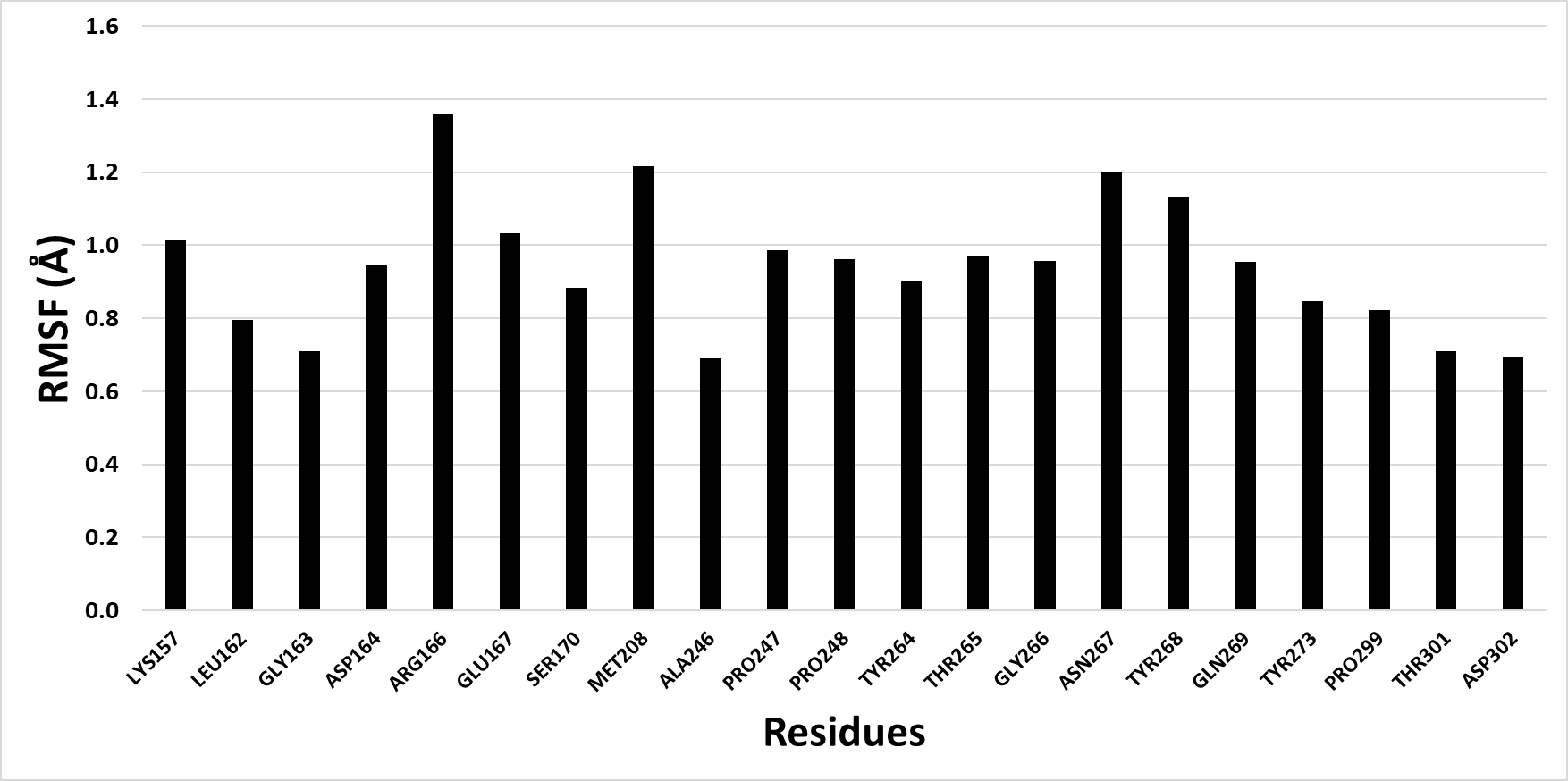


**Figure S3.** RMSD values (in Å) of the residues A) Y268 and B) K157 (to the right), for the SARS-CoV-2 PLpro structures utilized in cross-docking experiments involved in the structure-activity relationship model that exhibited the highest R². RMSD < 1.0 Å are represented in gray, RMSD ≥ 1.0 Å and < 2.0 Å are represented in blue, RMSD ≥ 2.0 Å and < 3.0 Å are represented in yellow, and RMSD ≥ 3.0 Å are represented in red. Conformations of each residue are represented to the left, colored differently according to their orientations.


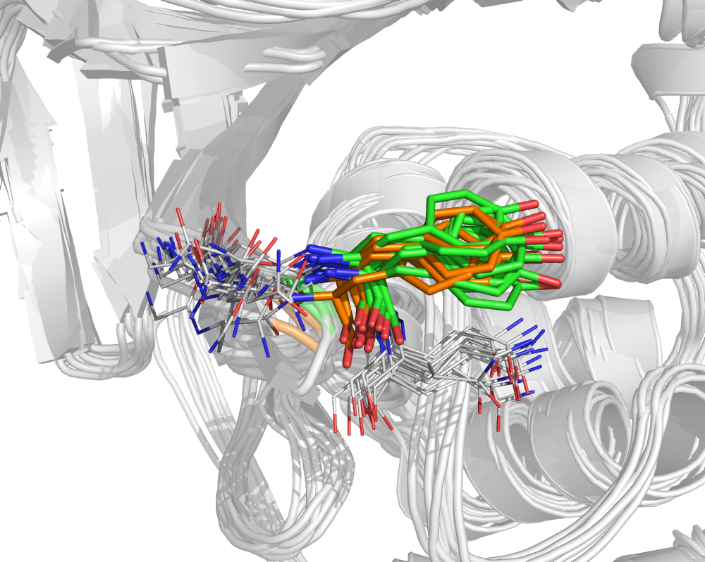


**A**


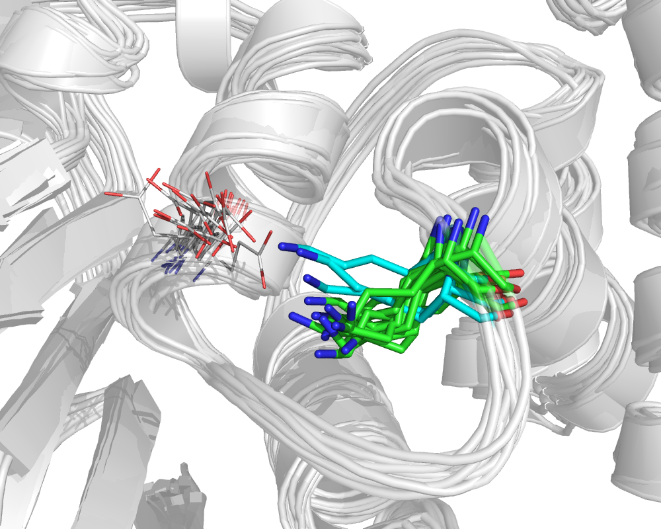


**B**

**Y268**

**K157**

**N267**

**Q269**

**E167**
